# Host-directed nanotherapy for the treatment and imaging of tuberculous meningitis

**DOI:** 10.7150/thno.125729

**Published:** 2026-03-03

**Authors:** Elizabeth W. Tucker, John Kim, Clara Erice, Anjali Sharma, Nerketa N. L. Damiba, Alvaro A. Ordonez, Javier Allende Labastida, Nirnath Sah, Filipa Mota, Patricia de Jesus, Rhea Saini, Kelly F. Schiaffino, Sanjay K. Jain, Rangaramanujam M. Kannan, Sujatha Kannan

**Affiliations:** 1 Center for Infection and Inflammation Imaging Research, Johns Hopkins University School of Medicine, Baltimore, MD 21287, USA.; 2 Center for Tuberculosis Research, Johns Hopkins University School of Medicine, Baltimore, MD 21287, USA.; 3 Department of Anesthesiology and Critical Care Medicine, Johns Hopkins University School of Medicine, Baltimore, MD 21287, USA.; 4 Center for Nanomedicine, Johns Hopkins University School of Medicine, Baltimore, MD 21287, USA.; 5 Department of Pediatrics, Johns Hopkins University School of Medicine, Baltimore, MD 21287, USA.

**Keywords:** dendrimer, tuberculous meningitis, microglia, PET imaging, host-directed therapy

## Abstract

**Rationale:**

Tuberculous meningitis (TB meningitis) is a devastating infection where the host immune response drives brain injury. Standard adjunctive corticosteroids often fail to prevent neurological sequelae or improve survival in many populations. Host-directed therapies that can cross the blood-brain barrier (BBB) and reduce neuroinflammation are urgently needed. We evaluated a hydroxyl-terminated polyamidoamine (PAMAM) dendrimer as a theranostic nanoplatform to visualize and treat microglia-mediated neuroinflammation in a young rabbit model of TB meningitis.

**Methods:**

A novel radiolabeled dendrimer (^124^I-dendrimer) was synthesized for noninvasive positron emission tomography (PET) imaging, with post-mortem gamma counting and fluorescent-labeled dendrimer (D-Cy5) confirming biodistribution. For therapy, rabbits with TB meningitis (i.e., infected) received weekly intravenous dendrimer-*N*-acetyl cysteine (D-NAC) or phosphate buffered saline (PBS). After two weeks, treatment efficacy was evaluated with longitudinal neurobehavioral scores and multimodal PET (^18^F-FDG for glucose metabolism, ^18^F-py-albumin for BBB integrity, and ^124^I-DPA-713 for microglial/macrophage activation). Post-mortem analyses included bacterial burden (colony-forming units [CFU]), cerebrospinal fluid (CSF) protein and cytokine levels, and brain immunohistochemistry for glial and white matter markers.

**Results:**

^124^I-Dendrimer demonstrated selective accumulation within brain lesions, co-localizing primarily with activated microglia. D-NAC significantly improved neurological outcomes and attenuated neuroinflammation and brain injury, even without antimicrobial therapy. Longitudinal PET imaging confirmed D-NAC efficacy, showing decreased neuroinflammation (^124^I-DPA-713) and improved BBB integrity (^18^F-py-albumin). Post-mortem analyses corroborated these findings, demonstrating that D-NAC reduced microglial inflammation and IL-17a levels, while improving myelination and BBB integrity.

**Conclusions:**

This study establishes D-NAC as a promising host-directed theranostic strategy for TB meningitis and supports the clinical potential of dendrimer nanoplatforms to diagnose and treat central nervous system infections.

## Introduction

Tuberculous meningitis (TB meningitis) is the most severe form of extrapulmonary tuberculosis (TB), disproportionately affecting vulnerable populations, including children and the immunocompromised 1, 2. The host immune system plays a crucial role in brain injury and contributes to its high mortality and morbidity rates, despite appropriate antimicrobial treatment 2, 3. TB meningitis is the only form of TB where the World Health Organization (WHO) recommends the use of corticosteroids as adjunctive therapy during standard treatment 4. However, corticosteroids are insufficient, only improving mortality in some cases, and failing to prevent neurological sequelae 5, 6. Outcomes vary due to host factors, such as leukotriene A4 hydrolase (LTA4H) genotype, tryptophan metabolism, and human immunodeficiency virus (HIV) status 7-10. Corticosteroids also have several limitations, such as systemic side effects like hypertension and hyperglycemia caused by off-target effects, and they may inhibit infection control while reducing drug penetration into the brain 11, 12. While other host-directed therapies (e.g., tumor necrosis factor [TNF] inhibitors, aspirin, and IL-1 receptor antagonists) are still under evaluation, concerns about non-specific immune suppression, off-target effects, and clinical effectiveness persist 13. Therefore, there is an urgent need for novel, precise host-directed therapies that can penetrate the blood-brain barrier (BBB) and regulate neuroinflammation without compromising infection control.

Activated microglia and astrocytes, primary immune cells of the central nervous system (CNS), play an important role in the robust immune response 11, 14, 15, while infiltration of peripheral immune cells further exacerbates injury 16. Microglia are preferentially infected by *Mycobacterium tuberculosis* 17, 18, with post-mortem studies showing dense, activated microglia co-localizing with brain lesions in both animal models and humans 11, 14, 15. *In vivo* positron emission tomography (PET)/computed tomography (CT) imaging using ^124^I-DPA-713, a translocator protein (TSPO) radiotracer more selective for activated microglia and macrophages, has revealed heterogeneous, lesion-specific neuroinflammation in TB meningitis 11, 15, 19-21. These findings highlight microglia as a key target for diagnosis and therapy in the assessment and treatment of neuroinflammation in TB meningitis.

*N*-acetyl cysteine (NAC), a glutathione precursor with anti-inflammatory and antioxidant properties, is a compelling host-directed therapy in TB because of its potential anti-mycobacterial effects 22. Small clinical studies in pulmonary TB report benefits of adjunctive NAC, including faster infection control, smaller cavity size, and higher glutathione peroxidase levels 23, 24. TB meningitis is characterized by significant neuroinflammation and oxidative stress, making NAC an appealing host-directed therapy candidate in this context 25. Unfortunately, NAC has poor bioavailability, limited brain penetration, and relies on the cysteine-glutamate antiporter (xCT) for uptake into glia. Due to limited intracellular delivery, free NAC increases extracellular glutamate and related excitotoxicity 26, 27, which already contributes to brain injury in TB meningitis patients 28.

To overcome current treatment limitations, we investigated dendrimers, small (~4 nm) nanoparticles that enable cell-targeted drug delivery, thereby reducing troublesome off-target side effects and enhancing drug efficacy. Hydroxyl-terminated polyamidoamine (PAMAM) dendrimers possess favorable biophysical properties that allow them to cross the impaired BBB, selectively accumulate in activated microglia and astrocytes, and deliver drugs intracellularly at the site of CNS pathology 29-32. NAC has been successfully conjugated to dendrimer (i.e., dendrimer-NAC [D-NAC]) and used in several models of neuroinflammation, including cerebral palsy, cardiac arrest, and Rett syndrome 30, 33-36. In these studies, D-NAC improved several markers of brain injury, including neurological outcome, oxidative stress, proinflammatory cytokines, and myelination 30, 33-36. Recently, a randomized, double-blind, phase 2a clinical trial evaluating D-NAC (OP-101®) in severe COVID-19 patients showed that it significantly reduced mortality and decreased markers of systemic inflammation and neurological injury 37. Importantly, it was well-tolerated in this first-in-human efficacy trial, demonstrating D-NAC's translational potential 37. Dendrimers represent a versatile platform with the capacity for both imaging and targeted drug delivery, positioning them as promising candidates for future theranostic applications. Indeed, a dendrimer-based PET imaging agent using the same dendrimer platform (OP-801^®^) is also in Phase 1/2 clinical testing for CNS neuroinflammation by Ashvattha Therapeutics (NCT05395624), showing dendrimer's potential as a diagnostic agent. In this study, we evaluated dendrimer's two complementary capabilities to visualize and treat neuroinflammation in our young rabbit model of TB meningitis.

## Materials and methods

### Experimental design

**Figure 1:**
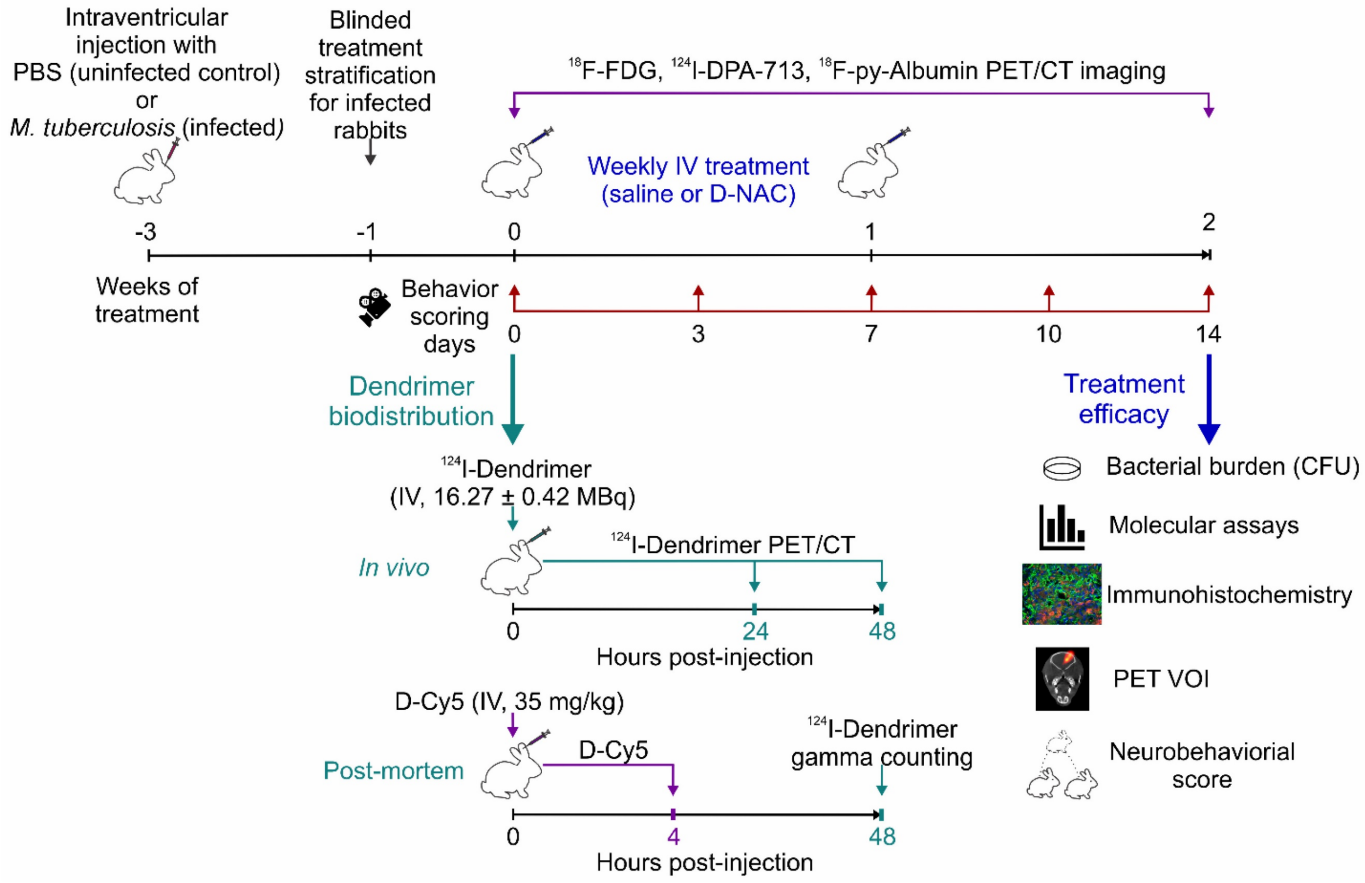
** Experimental Schematic.** Our study aimed to investigate dendrimer as a theranostic nanoplatform for visualizing and treating microglia-mediated neuroinflammation in a young rabbit model of TB meningitis. Dendrimer biodistribution was characterized using radiolabeled dendrimer (^124^I-dendrimer) for *in vivo* noninvasive positron emission tomography (PET) imaging and post-mortem *ex vivo* gamma counting, as well as fluorescent-labeled dendrimer (D-Cy5) for cellular localization with brain immunohistochemistry. Rabbits with TB meningitis (i.e., infected) were stratified to weekly intravenous treatments with phosphate buffered saline (PBS; saline-treated) or dendrimer-*N*-acetyl cysteine (D-NAC; D-NAC-treated), with all assessments performed by investigators blinded to treatment groups. Longitudinal neurobehavioral scores and multimodal PET imaging (^18^F-FDG, ^18^F-py-albumin, and ^124^I-DPA-713) were conducted throughout the study. After two weeks, treatment efficacy was evaluated through post-mortem bacterial burden (colony-forming units [CFU]), cerebrospinal fluid (CSF) protein and cytokine levels, and brain immunohistochemistry (microglial, astrocytic, and white matter markers), along with longitudinal neurobehavioral and multimodal PET analyses (volume of interests [VOIs]). Additional abbreviations: CT: computed tomography; IV: intravenous; MBq: megabecquerel.

To evaluate dendrimers as a theranostic nanoplatform, we first characterized their biodistribution using a radiolabeled dendrimer (^124^I-dendrimer) for *in vivo* noninvasive PET imaging and a fluorescent-labeled dendrimer (dendrimer-Cy5; D-Cy5) for cellular localization via post-mortem brain immunohistochemistry. To test therapeutic efficacy, we administered D-NAC monotherapy (i.e., without antimicrobials), recognizing that immunomodulators can increase bacterial burden, as previously seen with corticosteroids in TB meningitis 11. Rabbits with TB meningitis (i.e., infected rabbits) were stratified to receive weekly intravenous treatment (two doses total) with either phosphate buffered saline (PBS; saline-treated) or D-NAC (D-NAC-treated). Longitudinal neurobehavioral scoring and multimodal PET imaging (^18^F-FDG, ^124^I-DPA-713, ^18^F-py-albumin) were performed during treatment. After two weeks, treatment efficacy was evaluated using longitudinal measurements and post-mortem readouts of bacterial burden (colony-forming units [CFU]), cerebrospinal fluid (CSF) molecular assays (protein, cytokines), and brain histopathology (microglial, astrocyte, white matter, and amyloid plaque densities). The experimental schematic is shown in **Figure 1**. All protocols were approved by the Johns Hopkins University Biosafety, Animal Care and Use Committee (RB19M417), and Radiation Safety.

### Animal infection

We used our well-characterized young rabbit model of TB meningitis 11, 15, 19, 38, 39. In brief, male and female New Zealand White rabbits (Envigo Global Services, Inc., Denver, PA), obtained through in-house breeding, were inoculated intraventricularly with freshly reconstituted *M. tuberculosis* H37Rv (20 µL of 5.26 ± 1 log_10_ CFU) at 4-7 days old (i.e., infected rabbits). Uninfected control rabbits received an intraventricular injection of PBS (20 µL). The procedure was performed at the bregma (the intersection of the coronal and sagittal sutures) without fracturing or manipulating the cranium. All rabbits received topical lidocaine (4%; Ferndale IP Inc., Ferndale, MI) and dexmedetomidine (0.05-0.2 µg/g; Zoetis, Florham Park, NJ) for analgesia and sedation. Euthanasia was performed using intravenous pentobarbital sodium (120 mg/kg; VetOne, Boise, ID) at the designated time points.

### D-NAC synthesis

D-NAC was synthesized as previously described 30, 40. Briefly, NAC was conjugated to hydroxyl-PAMAM dendrimer (generation-4) using a disulfide bond only cleaved by intracellular concentrations of glutathione to allow for intracellular release as previously confirmed by characterization studies 30. D-NAC was purified, and previously established protocols confirmed the payload 41.

### Animal treatment

*M. tuberculosis* infection was established for three weeks before therapeutic intervention. Infected rabbits were stratified to receive D-NAC or saline treatment based on symptomatology, weight, sex, and brain lesions visualized noninvasively with CT and ^18^F-FDG imaging. The stratification of treatment groups was performed by investigators not involved in the study. Participating lab members were blinded to treatment conditions, including those who prepared and administered the treatment and performed data analysis. D-NAC (10 mg/kg NAC) or PBS (both clear liquids) was administered intravenously once weekly for two weeks (two total doses).

### Radiolabeled dendrimer (^124^I-dendrimer)

**Figure 2:**
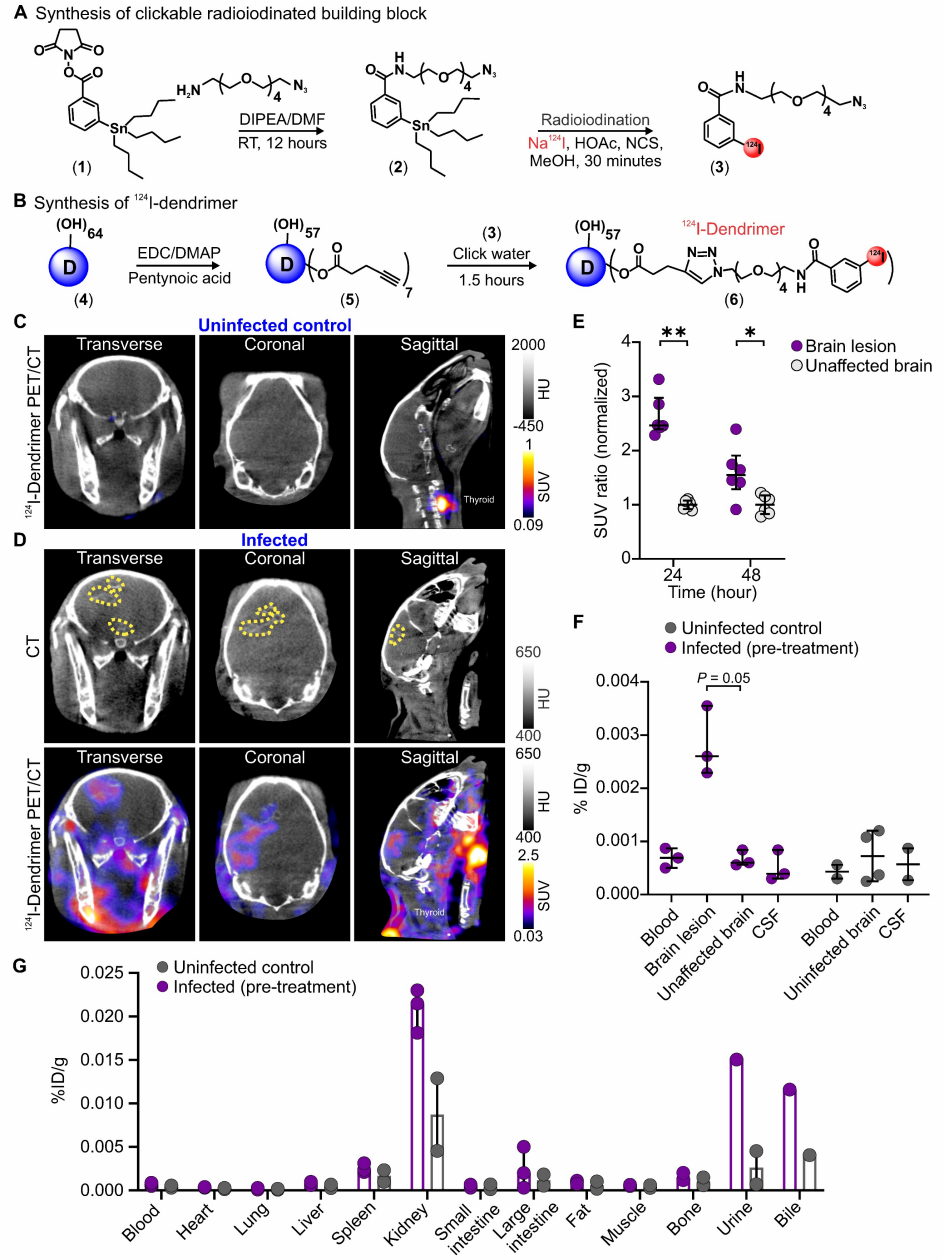
** Dendrimer penetrates the BBB and co-localizes with brain lesions.** (**A** and** B**) Radiolabeled dendrimer (^124^I-dendrimer) synthetic schematic. (**A**) The synthesis of ^124^I-dendrimer began with the construction of 3-(tributylstannyl)benzamide-tetraethyleneglycol-azide (compound **2**) from *N*-succinimidyl-3-(tri-n-butylstannyl) benzoate (STB, compound **1**) by reacting with azido-PEG5-amine. Compound **2** (clickable STB) was then radiolabeled by reacting with acetic acid (HOAc), sodium iodide I-124 (Na^124^I), and *N*-chlorosuccinimide (NCS) in methanol (MeOH) to obtain ^124^I-benzamide-tetraethyleneglycol-azide (compound **3**). (**B**) Compound **3** was further reacted with the alkyne-terminated dendrimer (D, compound **4**) using copper(I)-catalyzed azide-alkyne cycloaddition (CuAAC) click reaction to obtain ^124^I-dendrimer (compound **6**). (**C** and** D**) Representative transverse, coronal and sagittal ^124^I-dendrimer PET/CT imaged 24 hours (h) post-tracer injection and three weeks after injection with (**C**) PBS (uninfected control) or (**D**) *M. tuberculosis* (infected). The brain lesion hyperdensity (yellow dotted outline) was seen on CT (**D**, upper panel) and co-localized with ^124^I-dendrimer PET signal (**D,** lower panel) in the infected rabbit. (**E**) Serial ^124^I-dendrimer PET imaging presented standard uptake value (SUV) ratio (normalized to the mean of unaffected brain SUV) at 24 and 48 h post-tracer injection in infected rabbits. Each dot represents a volume of interest (VOI). PET-derived data include the following animals per time point: infected pre-treatment (*n* = 3, with 2 VOIs per animal, totaling 6 VOIs). (**F** and** G**) Post-mortem *ex vivo* biodistribution of ^124^I-dendrimer 48 h post-tracer injection using gamma counting (% injected dose [ID]/g) in the (**F**) CNS and (**G**) other organs/bodily fluids. Post-mortem data include the following animals per time point: uninfected control (*n* = 2) and infected pre-treatment (*n* = 3). Data are represented as median ± interquartile range (IQR). Statistical comparisons were made using Mann-Whitney tests (two-tailed, **E**, and one-tailed, **F**). *P < 0.05, **P < 0.01. Data are provided as a Source Data File. Additional abbreviations: DIPEA: *N*,*N*-diisopropylethylamine; DMAP: 4-dimethylaminopyridine; DMF: *N,N*-dimethylformamide; EDC: 1-ethyl-3-(3-dimethylaminopropyl)carbodiimide; HU: Hounsfield unit; RT: room temperature.

We developed a novel in-house radiosynthesis scheme to radiolabel dendrimers with I-124 using click chemistry, enabling noninvasive PET imaging for diagnostic purposes. **Figure 2** shows the synthesis scheme with detailed methods and validation described below and in **Supplementary Figures**. After in-house optimization, the protocol was transferred, and ^124^I-dendrimer was synthesized and purchased from 3D Imaging, LLC (Little Rock, AR) for animal imaging used in this study.

*Materials: N*-succinimidyl 3-(tri-*n*-butylstannyl) benzoate (STB, compound **1**) was purchased from Biosynth and used as received. Na^124^I was obtained from 3D Imaging, LLC (Little Rock, AR). Hydroxyl-PAMAM dendrimer (compound **4**) was sourced from Dendritech, Inc. (Midland, MI). All other reagents and solvents were obtained from Merck (Rahway, NJ) and used as received.

*Synthesis of compound **2**:* STB (compound **1;** 50 mg, 0.098 mmol) was reacted with an orthogonal azido-PEG5-amine (25.7 mg, 0.118 mmol) in the presence of *N*,*N*-diisopropylethylamine (DIPEA; 0.1 mL) and anhydrous *N*,*N*-dimethylformamide (DMF; 3 mL) for 12 hours (h) to produce a clickable STB (3-(tributylstannyl)benzamide-tetraethyleneglycol-azide; compound **2**). After completion of the reaction, the mixture was diluted with water (H_2_O) and extracted with ethyl acetate (20 mL x 3). The combined organic extracts were washed sequentially with H_2_O (20 mL x 3) and brine (20 mL x 2). The crude product was purified by column chromatography using methanol (MeOH) and dichloromethane (CH_2_Cl_2_), then evaporated under vacuum to obtain 72% purity. The proton nuclear magnetic resonance (^1^H NMR) clearly indicated the presence of polyethylene glycol (PEG) *H* (**Figure S1**). ^1^H NMR. (500 MHz, chloroform-d [CDCl_3_]) δ 7.83 (d, *J* = 1.9 Hz, 1H), 7.58 (d, *J* = 7.8 Hz, 1H), 7.51 (d, *J* = 7.1 Hz, 1H), 7.33 - 7.24 (m, 1H), 6.64 (s, 1H), 3.68 - 3.49 (m, 18H), 3.29 (t, *J* = 5.1 Hz, 2H), 1.52 - 1.36 (m, 6H), 1.26 (h, *J* = 7.3 Hz, 6H), 1.09 - 0.91 (m, 6H), 0.81 (t, *J* = 7.3 Hz, 9H).

*Synthesis of compound **3**:* Clickable STB (compound **2**; ~0.1 mg) was reacted with sodium iodide I-124 (Na^124^I; 314.5 MBq) in the presence of acetic acid (HOAc; 0.002 mL) and *N*-chlorosuccinimide (NCS, 0.025 mg) in MeOH (0.1 mL) to produce a radioiodinated clickable building block (^124^I-benzamide-tetraethyleneglycol-azide; compound **3**) using a minor modification of a previously published protocol 42. Compound **3** demonstrated a radiopurity of 84% by high-performance liquid chromatography (HPLC) (**Figure S2A**). The HPLC eluate of iodinated compound **3** was collected, diluted with H_2_O (10 mL), and purified using an activated C18 sep-pak cartridge. The cartridge was rinsed with H_2_O (10 mL) and dried under a nitrogen (N_2_) stream. A sodium sulfate (Na_2_SO_4_) drying tube (Alltech, catalog 219001) was connected at the end of the C18 cartridge, and the radiolabeled compound **3** was eluted with CH_2_Cl_2_ (2 mL). The purification yielded compound **3** (240.5 MBq) with 97% purity (**Figure S2B**).

*Synthesis of compound **5**:* Next, we modified the hydroxyl-PAMAM dendrimer (compound **4**) to produce an alkyne-terminated clickable dendrimer (compound **5**). We added pentynoic acid (11.58 mg, 0.118 mmol) to a stirring solution of hydroxyl-PAMAM dendrimer (compound **4**, 210 mg, 0.015 mmol) in anhydrous DMF (3 mL). 1-Ethyl-3-(3-dimethylaminopropyl)carbodiimide (EDC, 42.41 mg, 0.221 mmol) and 4-dimethylaminopyridine (DMAP, 27.03 mg, 0.221 mmol) were then added to the reaction mixture and stirred at room temperature for 12 h. It was subsequently dialyzed against DMF, followed by H_2_O. The dendrimer-containing aqueous solution was lyophilized to produce a solid white alkyne-terminated dendrimer (82% yield). The triplet at δ 4.03 ppm suggested the presence of an average of seven alkyne arms per dendrimer (**Figure S3**). ^1^H NMR. (500 MHz, dimethyl sulfoxide [DMSO]) δ 8.17-7.64 (D-internal amide *H*), 4.72 (s, D-OH), 4.03 (t, D-C*H_2_*-COO), 3.47-3.24 (m, D-C*H_2_*), 3.08-2.57 (m, D-C*H_2_ & linker H*), 2.47-2.34 (m, D-C*H_2_ & linker H*), 2.28-2.09 (m, D-C*H_2_ & alkyne H*).

*Synthesis of compound **6**:* Finally, ^124^I-dendrimer was synthesized by clicking compounds **3** and **5** using copper(I)-catalyzed azide-alkyne cycloaddition (CuAAC) click reaction. Compound **5** (150 µg) was dissolved in PBS (150 µL) and mixed with the radioiodinated compound **3**. Separately, fresh solutions of copper sulfate pentahydrate (1 mg/mL) and sodium ascorbate (1 mg/mL) were prepared in PBS. The copper sulfate pentahydrate solution (1 µL) and sodium ascorbate solution (2 µL) were combined and stirred for 1.5 h. Further purification was achieved by passing it through a 1 kDa Eppendorf filter, yielding 203.5 MBq of ^124^I-dendrimer with 92% purity confirmed by analytical radio-HPLC (**Figure S4**).

### PET/CT imaging and analysis

Live infected and uninfected control rabbits were imaged using sealed and transparent biosafety level-3 (BSL-3) compliant biocontainment devices, as previously reported 11, 15, 19, 38, 39, 43. All rabbits were initially anesthetized with dexmedetomidine (0.2 µg/g; Zoetis, Florham Park, NJ) for intravenous radiotracer injection, then maintained under an O_2_-isoflurane mixture. To perform multimodal imaging, radiotracer injections were done sequentially, with ^18^F (t_1/2_ ~109.8 minutes (min), short half-life radiotracers) imaging occurring one day before ^124^I (t_1/2_ ~4.2 days; long half-life radiotracers) to prevent contamination from residual radiotracer. ^18^F-FDG was purchased from Sofie Co. and ^124^I-DPA-713 was purchased from 3D Imaging, LLC. ^18^F-py-Albumin was synthesized in-house for animal use as previously described 11. The following imaging protocols were used: 15-min static PET performed 45 min post-injection of ^18^F-FDG (11.75 ± 1.02 MBq); 40-min dynamic PET performed immediately after injection of ^18^F-py-albumin (6.94 ± 0.95 MBq) and reconstructed as a static PET for analysis; 60-min static PET performed 24 h post-injection of ^124^I-DPA-713 (15 ± 1.1 MBq) as previously described 11, 15, 19, 38, 39, 43. PET imaging was initiated three weeks post-infection (before treatment initiation; week 0) based on the timing of symptomatology and gross pathology abnormalities in previous experiments 15, and repeated two weeks into treatment (week 2) to monitor treatment response.

A subset of live rabbits underwent noninvasive imaging with ^124^I-dendrimer (16.27 ± 0.42 MBq) before treatment initiation (week 0) to characterize dendrimer biodistribution. A 60-min static PET imaging protocol was performed 24 and 48 h after radiotracer administration. Imaging time points were chosen based on the known pharmacokinetics of the unlabeled dendrimer, which has significant brain penetration within four h and retention in the brain for several days 32.

Imaging was performed on a nanoScan PET/CT (Mediso, Arlington, VA) that automatically acquired and co-registered PET and CT images. VivoQuant 2020 (Invicro, Boston, MA) was used for PET analysis. Spherical three-dimensional volumes of interest (VOIs) were drawn in target brain regions, guided by CT as the anatomical reference, to quantify PET activity. PET signal was expressed as SUVmean (i.e., SUV) as previously reported 11, 15. To control technical variability (e.g., injected dose), an SUV ratio was calculated by normalizing uptake to an internal reference region. In infected rabbits, the SUV ratio was defined as brain lesion/contralateral unaffected brain, whereas in uninfected control rabbits, it was defined as right hemisphere/left hemisphere. All radiotracers were analyzed this way except ^124^I-dendrimer, whose biodistribution was evaluated in infected rabbits only using a normalized SUV ratio relative to the mean of the contralateral unaffected brain SUV in each animal. Treatment effects were determined using the SUV ratio fold change (post-treatment/pre-treatment) for individual VOIs in each animal, with the baseline scan serving as an internal control.

### *Ex vivo* dendrimer biodistribution

The same cohort of rabbits imaged by ^124^I-dendrimer PET/CT was euthanized 48 h post-injection. Tissues were collected, weighed, and radioactivity quantified by gamma counting (%ID/g) to evaluate *ex vivo* biodistribution as previously described 43. Another infected rabbit was injected with D-Cy5 (35 mg/kg) three weeks post-infection to localize the dendrimer within cells. It was euthanized after 4 h, perfused with PBS, and fixed in 4% paraformaldehyde (PFA) before staining and imaging (as described below).

### Neurobehavioral testing

A new, more sensitive neurobehavioral score, built on our previously reported score 15, was developed to quantify neuromotor deficits, such as deviations from normal postural and motor behavior, observed in infected rabbits. Neurobehavioral scoring began three weeks after intraventricular injection of *M. tuberculosis* (infected) or PBS (uninfected control). Open-field behavior was observed and videotaped from an aerial view for 2 min twice a week, starting before treatment initiation (week 0) and continuing through treatment (weeks 1 and 2). To reduce experimenter bias, the neurobehavior score included both automated and manual parts.

The automated part used the software Ethovision XT (Noldus), which has been previously used for rabbit neurobehavior 36. The following seven parameters were measured using Noldus: mean velocity (meter per second, [m/s]), distance traveled (m), mean absolute turn angle (degree), mean absolute angular velocity (degree per s), cumulative duration of movement (s), cumulative duration of mobility (s), and cumulative duration of normal body elongation (s). The scores from age-matched, uninfected control littermates were used to determine the average and standard deviation (SD) for each parameter, and the degree of deviation (i.e., number of SDs from the average) was used to score all rabbits. Noldus neurobehavioral scoring calculations and distribution are available in Supplementary Materials (**Supplementary Methods** and **Table S1**).

The manual part was scored by two researchers who were blinded to treatment conditions, and inter-rater agreement for each behavioral item was evaluated using observed agreement rates and quadratically weighted Cohen's kappa (κ) (**Supplementary Methods**). The following nine parameters were scored manually: clinical seizure, head tilt severity, limb paresis, hind limb paresis severity, front limb paresis severity, eye opening, hop, balance, and coat maintenance. A detailed description of all parameters is provided in Supplementary Materials (**Tables S2-3**). Neurobehavioral scores were nested to account for the effect of litter and serial scoring (with 1-2 scores per animal available per week of treatment) as described in the statistical analysis section.

### Post-mortem treatment response analysis

All rabbits were euthanized two weeks after treatment and perfused with PBS. CSF was obtained via cisterna magna, and organs were collected aseptically. Infected organs (brain, lung, spleen) were weighed, homogenized, and cultured on Middlebrook 7H11 selective plates (Becton Dickinson) to quantify bacterial burden (i.e., CFUs) as described previously 11, 19, 38, 39, 43.

*Molecular assays:* A subset of rabbits was used for molecular assays. CSF protein was measured using a BCA protein assay kit (ThermoFisher, catalog 23225). A rabbit antibody array (Abcam, catalog ab197459) was utilized to quantify levels of the following cytokines in the CSF: interleukin (IL)-17a, IL-21, IL-1α, TNF-α, IL-1β, IL-8, matrix metalloproteinase-9 (MMP-9), and macrophage inflammatory protein-1β (MIP-1β) with the following limit of detections (picogram [pg]/mL): IL-17a, 27.4-20,000; IL-21, 274.3-200,000; IL-1α, 13.7-10,000; TNF-α, 5.5-4,000; IL-1β, 1.4-1,000; IL-8, 0.3-200; MMP-9, 274.3-200,000; MIP-1β, 0.5-400. Samples were diluted 1:5, incubated following the manufacturer's protocol, and read on a microarray scanner (GenePix 4000B, Molecular Devices, LLC., San Jose, CA).

*Histopathology and immunohistochemistry:* Harvested brain tissue was fixed in 4% PFA and either placed in 30% sucrose for cryoprotection or paraffin-embedded. Paraffin-embedded tissue was sectioned at 5 µm thickness and stained with cresyl violet and Luxol fast blue (Abcam, catalog ab150675) to stain myelin, or with Congo red to stain amyloid plaques. Cryoprotected brain tissue was cryosectioned (20 µm thick) onto slides. Tissue was first blocked and permeabilized with 0.1% Triton-X solution and Donkey serum and then incubated with primary antibodies for microglia/macrophage-specific ionized calcium-binding adapter molecule 1 (Iba-1, 1:500 goat anti-Iba-1, Abcam, catalog ab107159) and/or glial fibrillary acidic protein (GFAP, 1:500 chicken anti-GFAP, Abcam, catalog ab4674). After washing with 1X tris-buffered saline (TBS) three times, tissue sections were incubated with the following secondary antibodies: AlexaFluor-488 donkey anti-chicken (1:1000, Jackson Immuno, catalog 703-545-155) and/or AlexaFluor-647 donkey anti-goat (1:1000, Invitrogen, catalog A-21447). The tissue was then washed in TBS and incubated in DAPI (ThermoFisher, catalog P36935). Fluorescently stained tissue sections were imaged using confocal microscopes (Nikon, C1si and Leica DMi8 inverted microscope with SP8 spectral confocal scanhead) using a Z stack (5 µm thick) and analyzed with HALO (Indica Labs) for quantification as previously described 11. Luxol fast blue density was quantified with Concentriq (Proscia, Philadelphia, PA).

### Statistical analysis

Data analysis was performed using Prism 10 (version 10.6.1, GraphPad) and R (version 4.3.3). Bacterial burden (CFU) data are presented as mean ± SD on a logarithmic scale (base 10). Molecular assay and weight data are presented as mean ± SD, while PET and immunohistochemical measurements are reported as median ± interquartile range (IQR). Statistical comparisons were conducted using the Mann-Whitney test (one- or two-tailed) for single comparisons, or the Kruskal-Wallis with Dunn's test for multiple comparisons of nonparametric data. Parametric data were analyzed by ANOVA followed by Tukey's multiple comparison test. To account for nested measurements (e.g., multiple behavior scores from the same rabbit within a litter over time), linear regression models fitted by generalized estimating equations (GEE) were applied to compare group means (β). *P* values ≤ 0.05 were considered statistically significant.

## Results

### Dendrimer targets activated microglia at sites of neuroinflammation

**Figure 3:**
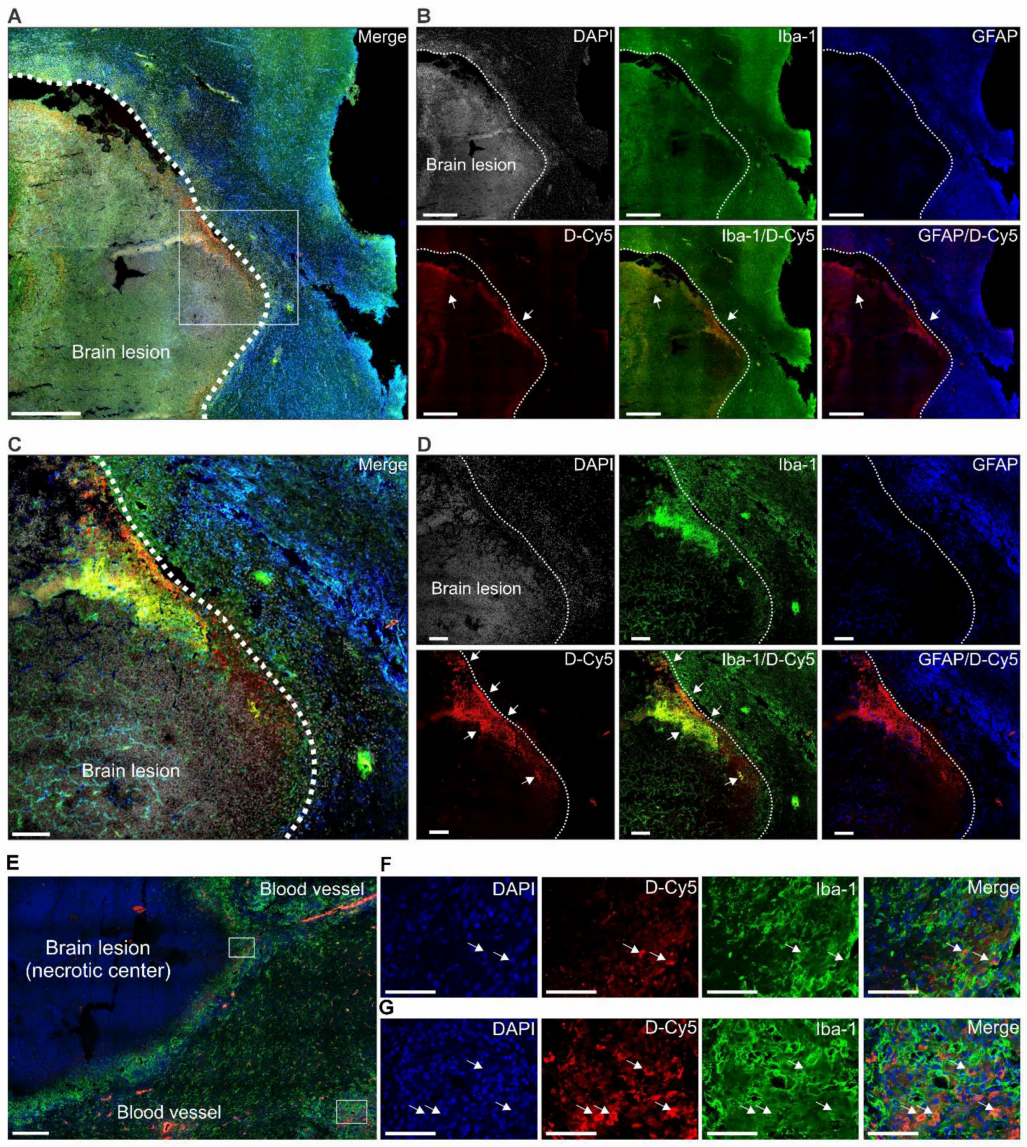
** Dendrimer co-localizes with activated microglia in and around brain lesions.** Representative confocal images of brain tissue obtained 4 h after injection of fluorescent-labeled dendrimer (D-Cy5) in an infected rabbit three-weeks post-infection and before treatment (*n* = 1). (**A** to** D**) Non-necrotic brain lesion (white dotted line) at low magnification. (**A**) Merged image and (**B**) individual panels of DAPI (gray, nuclei), Iba-1 (green, microglia), GFAP (blue, astrocyte), D-Cy5 (red, dendrimer), and corresponding Iba-1/D-Cy5, and GFAP/D-Cy5 overlays. Scale bar = 500 µm. The white box demarcates the area shown at high magnification in the merged image (**C**) and individual channels (**D**). Inset scale bar = 100 µm. White arrows indicate dendrimer co-localization with microglia, appearing yellow in the merged images (Iba-1/D-Cy5). Images in (**A**) and (**B**) were captured using Z stacks (5 µm) at 20x magnification, while (**C**) and (**D**) were acquired as a single plane at 20x. (**E** to** G**) Necrotic brain lesion shown at low magnification. (**E**) Merged image with DAPI (blue), D-Cy5 (red), and Iba-1 (green). Scale bar = 200 µm. White boxes outline regions of neuroinflammation shown at higher magnification in (**F**) the brain lesion rim and (**G**) a distal area of neuroinflammation. White arrows indicate co-localization of dendrimers within the cytoplasm of activated microglia. Inset scale bar = 50 µm. Images in (**E** to **G**) were captured as Z-stacks (5 µm) at 40x magnification.

The biodistribution of dendrimer was characterized using *in vivo*, *ex vivo,* and post-mortem analyses of ^124^I-dendrimer (radiolabeled dendrimer) (**Figure 2**) and D-Cy5 (fluorescent-labeled dendrimer) (**Figure 3**) in infected rabbits, three weeks after infection and before treatment was initiated. ^124^I-Dendrimer penetrated the BBB and localized within brain lesions, with a ~2.5-fold increase in the SUV in brain lesions compared with contralateral unaffected brain regions at 24 h (*P* < 0.01) (**Figure 2C-E**). This statistically significant difference persisted at 48 h (*P* < 0.05). *Ex vivo* ^124^I-dendrimer gamma counting at 48 h confirmed the PET imaging results and revealed a significant increase in radiotracer accumulation in brain lesions compared with unaffected brain regions of infected rabbits (3-fold increase, *P* = 0.05) (**Figure 2F**). These results demonstrate that ^124^I-dendrimer provides a robust signal-to-noise ratio, enabling the detection of neuroinflammatory lesions noninvasively. Additionally, ^124^I-dendrimer gamma counting verified that radiolabeled dendrimer behaved similarly to unlabeled dendrimer, with rapid renal excretion (**Figure 2G**). Post-mortem immunohistochemistry confirmed that D-Cy5 co-localized with activated microglia within non-necrotic brain lesions and around the rim of necrotic brain lesions but showed little accumulation in astrocytes (**Figure 3**).

### D-NAC attenuates microglial activation and restores BBB integrity

**Figure 4:**
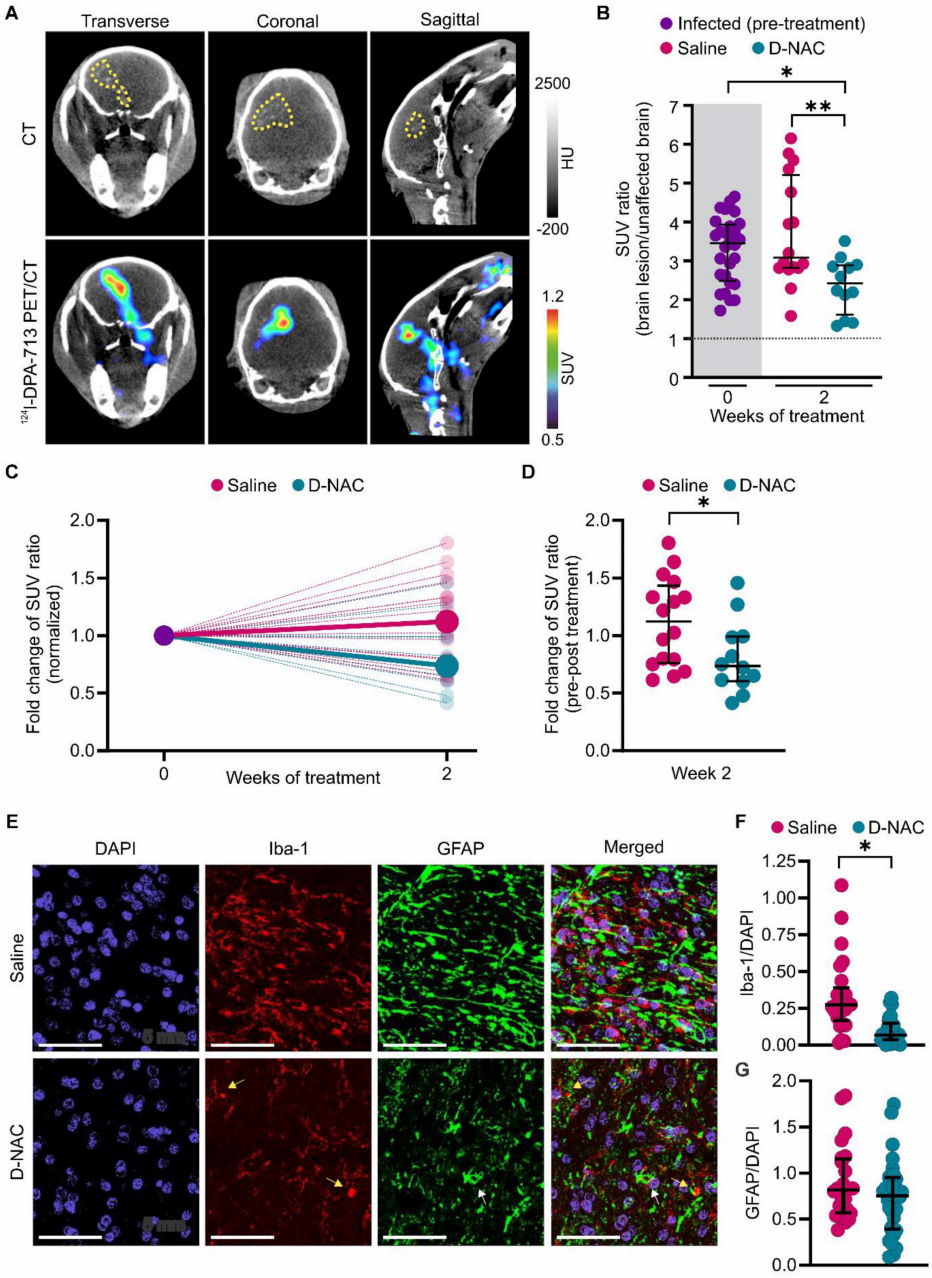
** D-NAC decreases microglial activation and density.** (**A**) Representative transverse, coronal, and sagittal ^124^I-DPA-713 PET/CT images of an infected rabbit imaged three weeks post-infection before treatment. The brain lesion (yellow dotted outline) is visible on CT (upper panel) and co-localizes with ^124^I-DPA-713 signal (lower panel). (**B**) PET-derived SUV ratios (brain lesion/unaffected brain) over treatment duration. Each dot represents an individual VOI. The pre-treatment time point (week 0) is shaded in gray. The dotted black line indicates a symmetric hemispheric signal observed in uninfected control animals (**Figure S5**). (**C**) PET-derived SUV ratio fold change normalized to week 0, shown for individual VOIs (small, light circles) and group medians (large, dark circles). (**D**) Comparison of SUV ratio fold change to pre-treatment levels for individual VOIs after two weeks of treatment. PET-derived data includes the following animals: infected pre-treatment (*n* = 11, with 2-3 VOIs per animal, totaling 28 VOIs), saline-treated (*n* = 6, with 2-3 VOIs per animal, totaling 16 VOIs), and D-NAC-treated (*n* = 5, with 2-3 VOIs per animal, totaling 12 VOIs). (**E**) Representative confocal images of microglia (Iba-1), astrocytes (GFAP), and nuclei (DAPI). Scale bar = 50 µm. (**F**) Quantification of microglial density (Iba-1^+^/DAPI^+^ cells) and (**G**) astrocyte density (GFAP^+^/DAPI^+^ cells). Immunohistochemical-derived data include the following animals: saline-treated (*n* = 3, with 2-3 sections per animal, totaling 24-25 regions) and D-NAC-treated (n = 4, with 2-3 sections per animal, totaling 28-32 regions). Data are represented as median ± IQR. Statistical comparisons were made using Kruskal-Wallis with Dunn's test (multiple comparisons, **B**), two-tailed Mann-Whitney test (single comparisons, **C**), and linear regression models fitted by generalized estimating equations (GEE) to compare group means (β) to account for nested data (**F** and **G**). *P < 0.05, **P < 0.01. Data are provided as a Source Data File.

After confirming that dendrimer localizes within brain lesions, we administered saline (saline-treated) or D-NAC (D-NAC-treated) intravenously once weekly for two weeks to infected rabbits to assess treatment efficacy (**Figure 1**). We previously showed that ^124^I-DPA-713, a clinically translatable TSPO radiotracer 44 with greater specificity for activated microglia and macrophages, co-localizes with brain lesions in animal models, including our rabbit model of TB meningitis 11, 15, 19. Therefore, we tested its ability to monitor the response to our microglia-specific, host-directed nanotherapy. We confirmed that ^124^I-DPA-713 exhibited a symmetric distribution in the uninfected control brain (SUV ratio ~1) but co-localized with brain lesions in infected rabbits, demonstrating lesion-specific microglial activation (**Figure 4A** and **Figure S5**). D-NAC treatment significantly reduced the ^124^I-DPA-713 SUV ratio (brain lesion/unaffected brain) compared to both infected rabbits pre-treatment (*P* < 0.05) and the saline-treated group (*P* < 0.01) (**Figure 4B**). Analysis of individual lesional changes before and after treatment further demonstrated divergent responses between treatment groups. While the ^124^I-DPA-713 signal attenuated in most brain lesions following D-NAC treatment, the signal continued to increase in most saline-treated rabbits (**Figure S6**). Accordingly, the median SUV ratio fold change increased in saline-treated rabbits, whereas it decreased in D-NAC-treated rabbits when normalized to their pre-treatment ^124^I-DPA-713 signal (*P* < 0.05) (**Figure 4C-D**). Post-mortem brain immunohistochemistry confirmed decreased neuroinflammation, showing a significant reduction in microglial density in D-NAC-treated rabbits compared with saline-treated rabbits (*P* < 0.05), with an appearance resembling uninfected control brain tissue (**Figure 4E-F** and **Figure S7**). Conversely, astrocyte density was unchanged following D-NAC treatment (*P* = 0.089) (**Figure 4G**). Overall, these findings support dendrimer's ability to deliver targeted therapy to activated microglia, as previously described in several models of neuroinflammation 30, 32-36.

**Figure 5:**
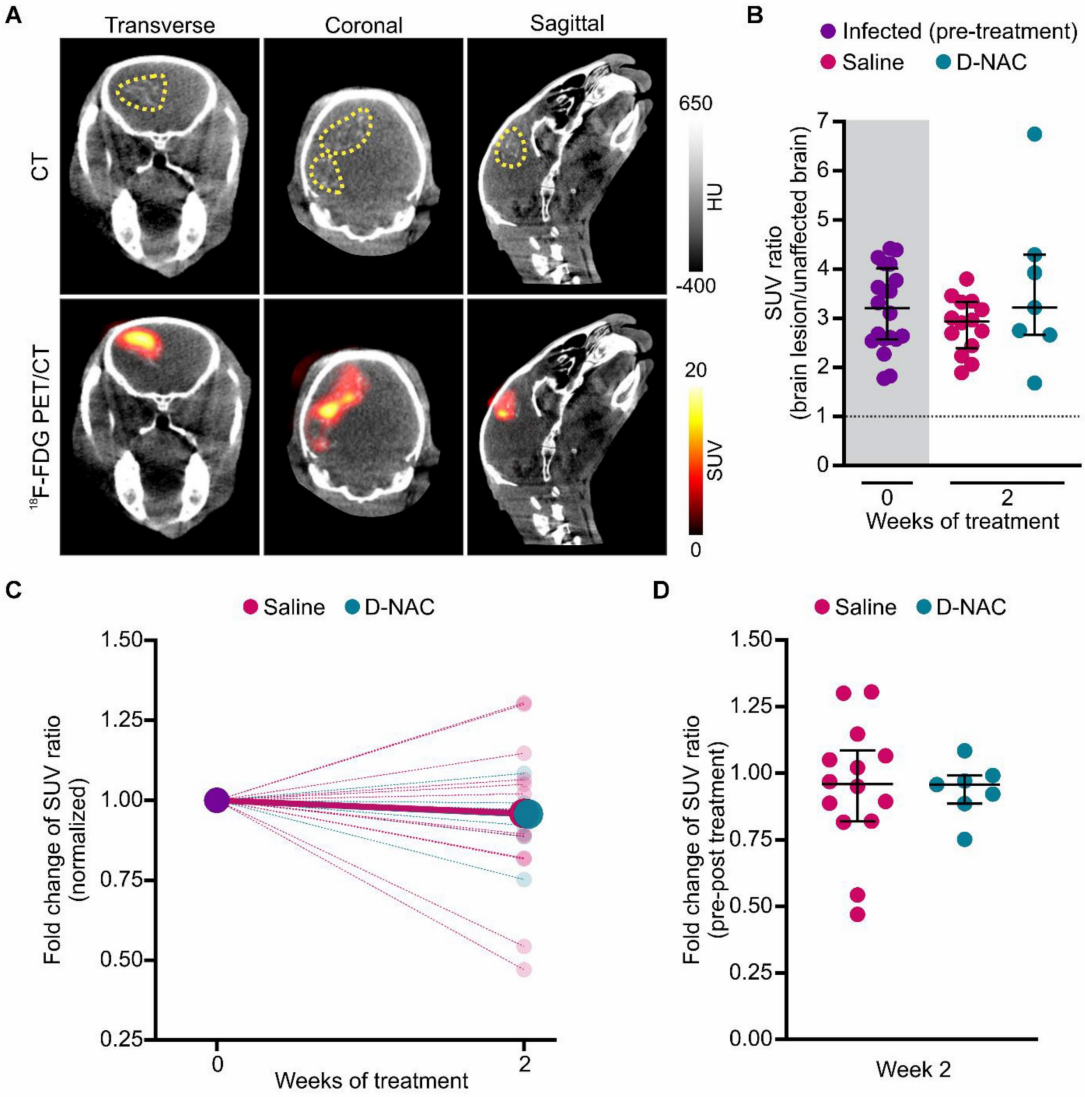
**^18^F-FDG co-localizes with brain lesions, but cannot assess treatment efficacy.** (**A**) Representative transverse, coronal, and sagittal ^18^F-FDG PET/CT images of an infected rabbit imaged three weeks post-infection before treatment. The brain lesion hyperdensity (yellow dotted outline) is seen on CT (upper panel) and co-localizes with ^18^F-FDG signal (lower panel). (**B**) PET-derived SUV ratios (brain lesion/unaffected brain) over treatment duration. Each dot represents an individual VOI. The pre-treatment time point (week 0) is shaded in gray. The dotted black line indicates a symmetric hemispheric signal observed in uninfected control animals (**Figure S8**). (**C**) PET-derived SUV ratio fold change normalized to week 0, shown for individual VOIs (small, light circles) and group medians (large, dark circles). (**D**) Comparison of SUV ratio fold change to pre-treatment levels for individual VOIs after two weeks of treatment. Data include the following animals: infected pre-treatment (*n* = 8, with 2-3 VOIs per animal, totaling 20 VOIs), saline-treated (*n* = 5, with 2-3 VOIs per animal, totaling 14 VOIs), and D-NAC-treated (*n* = 3, with 2-3 VOIs per animal, totaling 7 VOIs). Data are represented as median ± IQR, and statistical comparisons were made using Kruskal-Wallis with Dunn's test (multiple comparisons, **B**) and two-tailed Mann-Whitney test (single comparisons, **C**). Data are provided as a Source Data File.

Given the extensive utilization of ^18^F-FDG in patients with TB meningitis and animal models to identify brain lesions 11, 20, 38, 43, 45, we investigated its ability to monitor treatment-related neuroinflammatory changes (**Figure 5**). Although ^18^F-FDG exhibited a symmetric distribution in the uninfected control brain (**Figure S8**) and co-localized with brain lesions in infected rabbits (**Figure 5A**), it did not reflect the therapeutic attenuation of microglial activation that was captured by ^124^I-DPA-713 and confirmed by post-mortem immunohistochemistry (**Figure 5B-D** and **Figure S9**). Consequently, while ^18^F-FDG is an effective tool for lesion identification, our results suggest it lacks sufficient specificity to monitor microglia-driven inflammatory activity in this early timeframe. This likely reflects the fact that glucose metabolism may not be substantially changed at this stage of disease or may remain elevated in both pro-inflammatory and anti-inflammatory microglial phenotypes.

**Figure 6:**
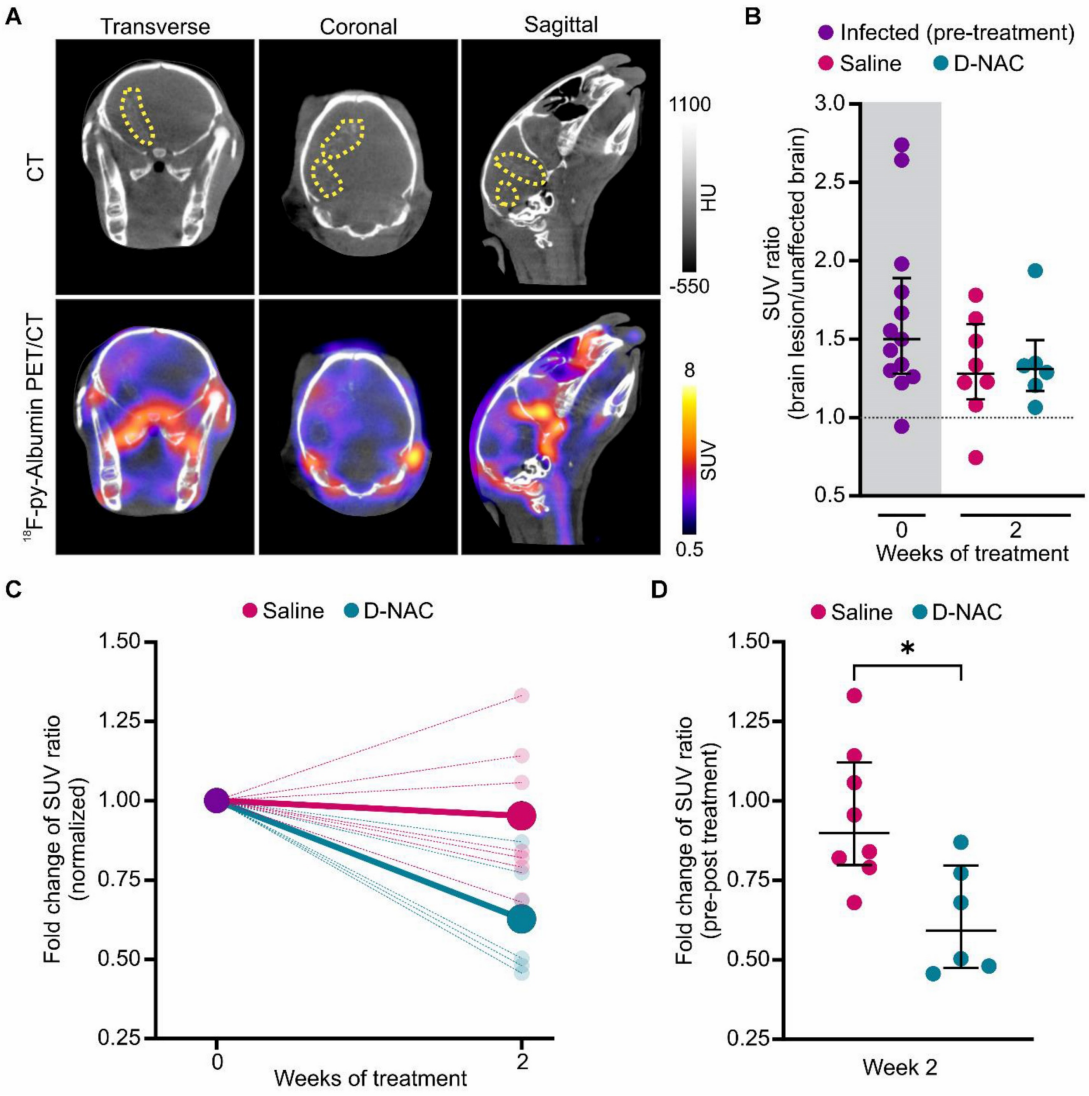
**^18^F-py-Albumin demonstrates healing BBB with D-NAC.** (**A**) Representative transverse, coronal, and sagittal ^18^F-py-albumin PET/CT images of an infected rabbit imaged three weeks post-infection before treatment. The brain lesion (yellow dotted outline) is seen on CT (upper panel) and co-localizes with ^18^F-py-albumin signal (lower panel). (**B**) PET-derived SUV ratios (brain lesion/unaffected brain) over treatment duration. Each dot represents an individual VOI. The pre-treatment time point (week 0) is shaded in gray. The dotted black line indicates a symmetric hemispheric signal observed in uninfected control animals (**Figure S10**). (**C**) PET-derived SUV ratio fold change normalized to week 0, shown for individual VOIs (small, light circles) and group medians (large, dark circles). (**D**) Comparison of SUV ratio fold change to pre-treatment levels for individual VOIs after two weeks of treatment. Data include the following animals: infected pre-treatment (*n* = 5, with 2-3 VOIs per animal, totaling 13 VOIs), saline-treated (*n* = 3, with 2-3 VOIs per animal, totaling 8 VOIs), and D-NAC-treated (*n* = 2, with 3 VOIs per animal, totaling 6 VOIs). Data are represented as median ± IQR, and statistical comparisons were made using Kruskal-Wallis with Dunn's test (multiple comparisons, **B**) and two-tailed Mann-Whitney test (single comparisons, **C**). *P < 0.05. Data are provided as a Source Data File.

In addition to intracerebral inflammation, TB meningitis is characterized by impaired BBB integrity, evidenced by elevated CSF protein levels and contrast enhancement on magnetic resonance imaging (MRI), as demonstrated in our TB meningitis model 15, 38. Building on previous studies where ^18^F-py-albumin PET captured lesion-specific BBB disruption and healing following two weeks of antimicrobial and corticosteroid treatment 11, we utilized longitudinal ^18^F-py-albumin imaging to evaluate D-NAC's therapeutic impact on BBB integrity (**Figure 6**). As expected, infected rabbits had baseline BBB compromise with increased lesion-specific ^18^F-py-albumin signal compared to the symmetric distribution seen in the uninfected control rabbits (**Figure 6A-B** and **Figure S10**). Following D-NAC treatment, we observed a statistically significant decrease in ^18^F-py-albumin uptake, with a reduced median SUV ratio fold change compared to saline-treated rabbits when normalized to their pre-treatment ^18^F-py-albumin signal (*P* < 0.05) (**Figure 6C-D** and **Figure S11**). These imaging findings, which mirror the clinical recovery of BBB integrity, indicate that D-NAC monotherapy is sufficient to restore BBB function.

### Microglia-targeted D-NAC therapy ameliorates neurological deficits and preserves myelination

**Figure 7:**
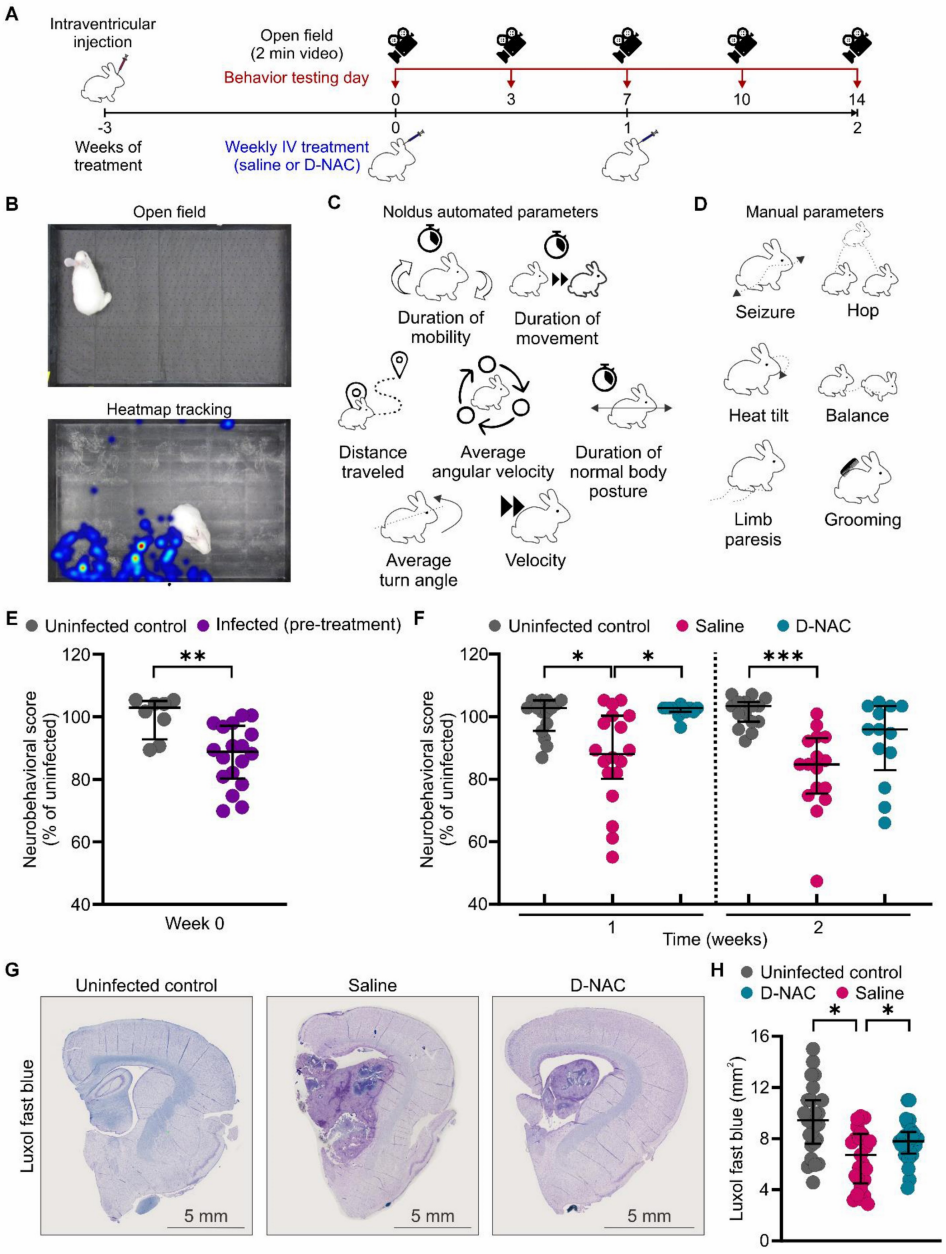
** D-NAC attenuates neurobehavioral deficits and improves myelination.** (**A**) Experimental schematic of longitudinal neurobehavioral scoring using two-min videos obtained before treatment initiation (week 0) and twice weekly during treatment (weeks 1 and 2). (**B**) Representative video frame of open field arena (upper panel) and corresponding heatmap of rabbit movement over the recording duration (lower panel), generated using Noldus software. (**C** and** D**) Components of neurobehavioral score with (**C**) Noldus automated parameters and (**D**) manual parameters. (**E**) Neurobehavioral score (% of uninfected control score) before treatment initiation. (**F**) Longitudinal neurobehavioral score (% of uninfected control score) after one and two weeks of treatment. Each dot represents an individual score. Neurobehavioral data include the following animals, with 1-2 scores per animal: uninfected control (*n* = 8), infected pre-treatment (*n* = 18), saline-treated (*n* = 9), and D-NAC-treated (*n* = 7). (**G**) Representative bright field image of Luxol fast blue (myelin) with cresyl violet co-stain. (**H**) Myelination density quantification (mm^2^). Immunohistochemical data include the following animals, each with 4-5 sections: uninfected control (*n* = 5), saline-treated (*n* = 5), and D-NAC-treated (*n* = 5). Data are represented as median ± IQR. Statistical comparisons were made using two-tailed Mann-Whitney for single comparisons (**E**) and linear regression models fitted by GEE to compare group means (β) to account for nested data (**F** and** H**). *P < 0.05, **P < 0.01, ***P < 0.001. Data are provided as a Source Data File.

We have previously demonstrated that infected rabbits reach developmental milestones more slowly than uninfected control rabbits and develop neurological deficits, such as head tilt, abnormal gait, and hindlimb weakness, by three weeks of infection 15. To monitor treatment response, we performed longitudinal assessments using serial neurobehavioral videos analyzed with our newly developed, reliable scoring system expanded from our previous work 15 (**Figure 7A-D**). At baseline, infected rabbits exhibited a statistically significant decrease in neurobehavioral scores compared to their uninfected control, age-matched littermates (14% reduction; *P* < 0.01) (**Figure 7E**).

Rapid functional recovery was observed following the first dose of D-NAC (week 1), with D-NAC-treated rabbits exhibiting significantly better neurobehavioral scores than the saline-treated rabbits (*P* < 0.05) (**Figure 7F**). Notably, none of the D-NAC-treated rabbits had scores below those of their uninfected control littermates, whereas 44.4% of saline-treated rabbits did (**Figure S12**). After two doses (week 2), the neurological deficits continued to worsen in saline-treated rabbits, which maintained statistically significant differences compared to their uninfected control littermates (*P* < 0.001) (**Figure 7F**). In contrast, D-NAC-treated rabbits showed no significant differences relative to uninfected control rabbits, indicating a near-normal functional status at this time point. By the second week, only 38.5% of the D-NAC-treated rabbits remained below those of their uninfected control littermate scores, compared to 68.8% of saline-treated rabbits with worse scores than their uninfected control littermates (**Figure S12**).

Interestingly, we found no sex-based differences in neurobehavioral scores (**Figure S13**). Weight gain was monitored as an important clinical indicator of systemic disease burden, and both saline-treated and D-NAC-treated infected rabbits gained less weight and were smaller than uninfected control rabbits (**Figure S14**). This suggests that, while the systemic impact of the infection persisted, the focal neurological deficits, driven by neuroinflammation, were effectively and selectively ameliorated by D-NAC.

This behavioral recovery was corroborated by post-mortem analysis of myelination, which had previously improved with D-NAC treatment in other models of neuroinflammation 30. We measured myelin density using Luxol fast blue staining and found it was significantly lower in saline-treated rabbits compared to uninfected rabbits (*P* < 0.05) (**Figure 7G-H**). Critically, myelin density was restored in D-NAC-treated rabbits, establishing a structural basis for the observed functional neurological recovery.

### D-NAC modulates brain injury markers without exacerbating bacterial burden

**Figure 8:**
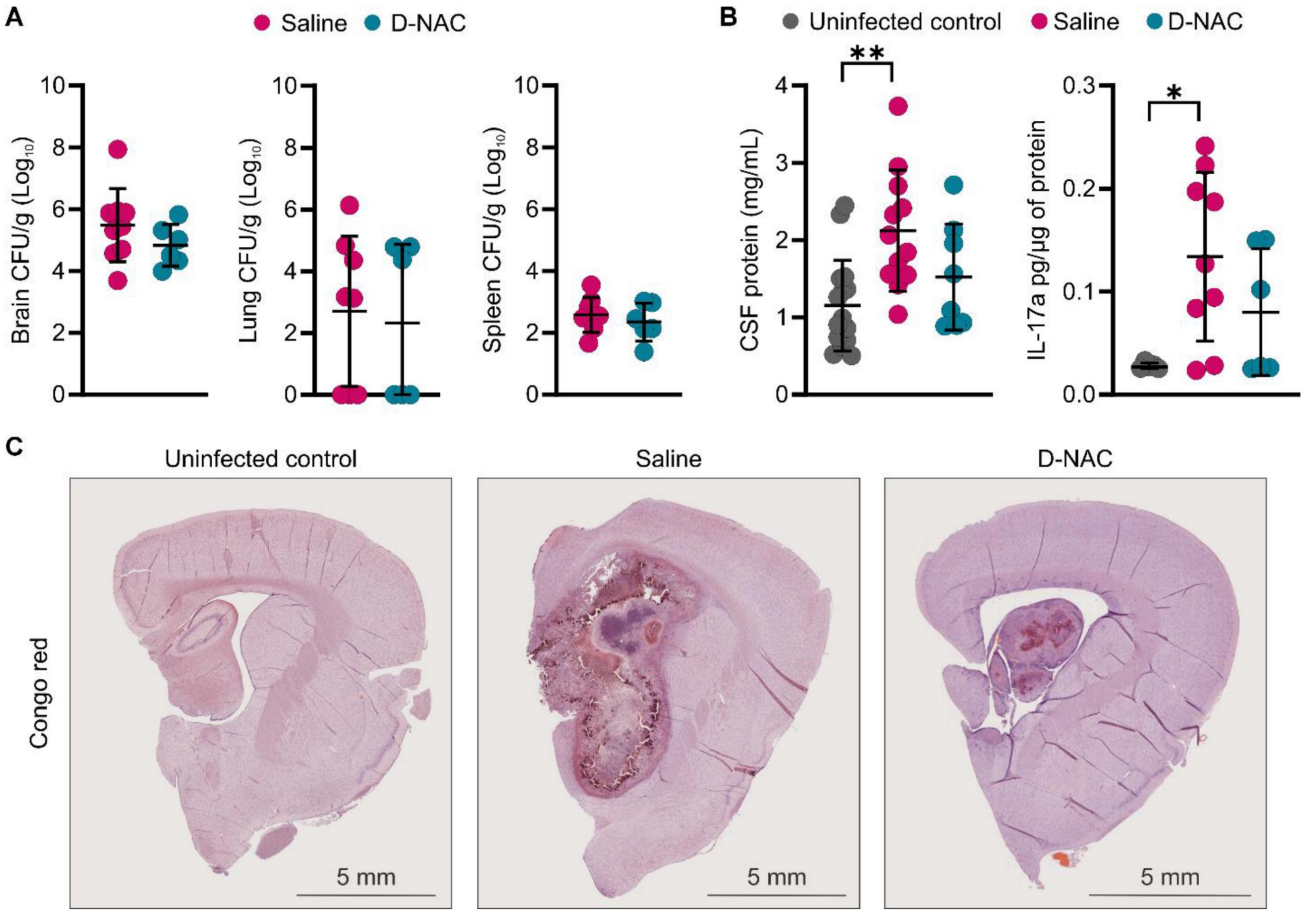
** D-NAC improves post-mortem inflammatory and brain injury markers without affecting bacterial burden.** All samples were collected in age-matched uninfected control rabbits or infected rabbits after two weeks of treatment with saline (saline-treated) or D-NAC (D-NAC-treated). (**A**) Bacterial burden (colony forming units [CFU]/g tissue) in the brain (left), lung (middle), and spleen (right). Bacterial burden data include the following animals: saline-treated (*n* = 8-9) and D-NAC-treated (*n* = 6. (**B**) Cerebrospinal fluid (CSF) protein (left) and IL-17a (right). CSF data include the following animals: uninfected control (*n* = 5-16), saline-treated (*n* = 9-12), and D-NAC-treated (*n* = 6-8). (**C**) Representative bright field images of Congo red (amyloid plaques). Data are represented as mean ± SD with CFU (**A**) on a logarithmic scale (base 10). Statistical comparisons were made using a two-tailed *t*-test (single comparisons, **A**) and ANOVA with Tukey's (multiple comparisons, **B**). *P < 0.05, **P < 0.01. Data are provided as a Source Data File.

While host-directed therapies can help by reducing pathological immune responses, they can also impair the host's ability to control bacterial growth. This poses a serious risk for infections like TB meningitis, as it may lead to higher bacterial loads. Based on recent data showing increased bacterial burden with corticosteroid therapy in a mouse model of TB meningitis, we measured bacterial burden across several compartments in our rabbit model. We observed no difference in bacterial load (expressed as CFU) in the brain, lung, or spleen between rabbits treated with saline and those treated with D-NAC, confirming that D-NAC did not impair the immune system's ability to control *M. tuberculosis* replication (**Figure 8A**).

We next examined post-mortem changes in markers of BBB integrity and brain injury following saline or D-NAC treatment. CSF analysis showed that saline-treated rabbits had significantly higher protein (*P* < 0.01) and IL-17a (*P* < 0.05) levels, while levels in D-NAC-treated rabbits decreased to values similar to those of uninfected rabbits (**Figure 8B**). No significant differences in other cytokines were observed between uninfected and infected rabbits at this time point, except for IL-1α, which was significantly increased in infected rabbits and remained unchanged by D-NAC treatment (**Figure S15**). Given the chronic nature of TB meningitis, we qualitatively assessed for signs of neurodegeneration by staining for amyloid deposits using Congo red. The uninfected control rabbits showed no evidence of amyloid staining, as expected for the juvenile brain (**Figure 8C**). Amyloid deposition was readily observed in the brains of infected rabbits, indicating that TB meningitis leads to neurodegenerative changes in the brain. Due to the limited sample size, these Congo red findings are presented as qualitative observations, as we were unable to compare plaque density between saline- and D-NAC-treated groups.

## Discussion

TB meningitis remains a devastating disease with high mortality and long-term neurological sequelae, especially in children. Despite advancements in antimicrobial therapy, tempering the hyperinflammatory immune response without inducing secondary immune suppression remains a critical treatment priority 2. Many recent and ongoing clinical trials have evaluated adjunctive host-directed therapies, such as corticosteroids, aspirin, doxycycline, and TNF inhibitors (e.g., INTENSE-TBM, LASER-TBM, INSHORT, DIRECT, and the ACT HIV trial). To date, clinical outcomes remain mixed, and no host-directed therapy has been proven to consistently decrease mortality and neurological deficits across all patient populations, particularly those with HIV co-infection 6, 10, 46-50. These suboptimal results are likely due to multiple factors, including poor CNS drug delivery, difficulty in selectively targeting sites of injury, and harmful off-target side effects like systemic immune suppression. In this study, we show that a theranostic dendrimer platform can address these fundamental limitations. *In vivo* PET imaging with ^124^I-dendrimer demonstrated lesion-specific accumulation, and post-mortem immunohistochemistry confirmed cellular localization predominantly within activated microglia. Furthermore, we showed that just two weekly systemic doses of D-NAC (10 mg/kg NAC) were sufficient to provide significant therapeutic benefits without exacerbating bacterial burden, even without antimicrobial therapy.

The clinical potential of D-NAC is driven by its cell-targeted delivery, which offers a significant advantage for host-directed therapies in CNS diseases by enabling selective uptake into neuroinflammatory cells. Previous studies have demonstrated that conjugating NAC to the dendrimer increases its potency by approximately 100-fold compared to free NAC, allowing for lower and less frequent dosing to achieve therapeutic effect 30, 37. This was clinically validated in a COVID-19 trial, where a single dose of D-NAC (containing only 0.3-1.3 mg/kg of NAC) provided lasting therapeutic benefits, with improved mortality and significantly reduced neurological injury markers 37. The weekly dosing schedule used in our model suggests a path to simplify the complex treatment regimen for TB meningitis. Although we used intravenous administration, oral D-NAC is available and has been shown to decrease microglial activation and preserve myelination in a mouse model of necrotizing enterocolitis 51. While more research is needed, weekly oral D-NAC could be a promising adjunctive therapy for TB meningitis, especially in resource-limited settings where simplified administration is vital.

Our synthesis and characterization of ^124^I-dendrimer demonstrated selective accumulation in regions of neuroinflammation, especially within activated microglia. This new synthesis provides proof-of-concept that dendrimer-PET could serve as a noninvasive diagnostic tool to identify sites of CNS injury and potentially guide personalized therapy. Despite the widespread use of TSPO-PET in clinical studies of neuroinflammation, neuropsychiatric disorders, and neurodegeneration 20, 52, 53, its utility is complicated by genetic polymorphisms (e.g., in the *TSPO* gene) that alter binding affinities and cause significant patient variability 54. Dendrimer-PET provides a clear advantage over TSPO-PET for clinical translation. Unlike TSPO radiotracers, dendrimer uptake occurs through receptor-independent fluid-phase endocytosis in activated glia 55, making it less affected by genetic differences. Dendrimer-PET also offers a unique theranostic platform in which the same dendrimer scaffold enables both radiolabeling for imaging and drug conjugation for targeted therapy. While radiolabeled dendrimers have gained attention in oncology, their use in infectious diseases remains largely unexplored 56. Notably, ^18^F-labeled dendrimers have demonstrated superior sensitivity compared to TSPO-PET for detecting neuroinflammation in a mouse model of Alzheimer's disease 57. Although further studies are necessary to confirm whether dendrimer-PET can reliably monitor treatment response with minimal patient variability, an ongoing first-in-human study using the same dendrimer (OP-801®) for non-infectious neuroinflammation (NCT05395624) will shed light on its potential for imaging neuroinflammatory conditions.

Using ^124^I-DPA-713, a well-established PET ligand for detecting TSPO expression as a marker of microglial activation, we showed that D-NAC treatment led to a significant decrease in ^124^I-DPA-713 signal that correlated with decreased microglial activation on immunohistochemistry. While TSPO is expressed in several cell types 58, our previous work demonstrated a predominant increase in microglial density and activation around brain lesions where ^124^I-DPA-713 co-localizes 15. This suggests that most of the ^124^I-DPA-713 signal in our model reflects microglial activation, and the reduction observed after D-NAC treatment indicates it effectively targeted activated microglia. Although ^18^F-FDG has been widely used to identify brain lesions as a surrogate marker of inflammation due to its widespread availability 11, 38, 43, 45, we found that longitudinal imaging did not correlate well with changes in microglial activation in our model. This is not surprising, as ^18^F-FDG measures regional glucose metabolism and lacks cell-type specificity. Indeed, similar divergences between ^18^F-FDG and TPSO-PET ligands have been previously reported 59. While we did not utilize ^124^I-dendrimer to assess treatment response in this study, previously published data suggest that dendrimer-PET may offer superior sensitivity to TSPO-PET in detecting subtle changes in microglial activation 57. Because the imaging tracer and the therapeutic D-NAC share the same dendrimer scaffold, this platform could function as a truly integrated theranostic agent. Together, these findings highlight the potential of a dendrimer-based platform to selectively image and deliver D-NAC to the activated microglia in TB meningitis.

To our knowledge, this study provides the first evidence supporting D-NAC as a potential host-directed therapy for TB meningitis. Notably, D-NAC therapy improved the neurological deficits that developed in infected rabbits, highlighting its potential to limit brain injury where traditional host-directed therapies have often fallen short 5, 6, 10, 47. These therapeutic effects were observed within a two-week treatment period, which represents the critical window when TB meningitis mortality is highest 60. Half of hospitalized deaths occur within two weeks of admission 61, therefore demonstrating significant functional and structural recovery during this timeframe is clinically paramount. Post-mortem analysis supported these findings, showing preservation of myelination after D-NAC treatment, similar to improvements reported in other experimental models 30. Importantly, these neurobehavioral improvements occurred without increasing the bacterial burden, a substantial limitation of corticosteroids 11. We also observed significant amyloid deposition in infected rabbits, resembling the pathology seen in neurodegenerative diseases, such as Alzheimer's disease, where chronic microglial activation drives disease progression 62, 63.

In addition to improving functional outcomes, D-NAC decreased BBB permeability, as evidenced by reductions in CSF protein and ^18^F-py-albumin signal within brain lesions. These results are comparable to changes observed with intensive antimicrobial treatment (with or without corticosteroids) 11, 38. Similar to other treatment studies, the changes in cytokine levels were mixed 64, 65. We observed a significant improvement in IL-17a following D-NAC treatment, aligning with patient data showing that IL-17a, predominantly produced by T helper 17 (Th17) cells to recruit neutrophils, is significantly higher in active TB than in latent infection 65, 66. Th17 cells have been linked to multiple sclerosis, a chronic CNS autoimmune disease, with their interactions with microglia and astrocytes contributing to disease progression 67, 68. Our findings may suggest that D-NAC modulates the crosstalk between microglia and Th17 cells. While IL-1β, the main secreted form of IL-1, is often elevated in TB meningitis 65, 69, we did not detect a significant increase in our model. Instead, IL-1α, the primary intracellular form, was elevated and remained unaffected by D-NAC treatment. While the role of IL-1α in TB meningitis has not been widely studied, a murine stroke model showed that IL-1α was neuroprotective, reducing infarct size and improving functional outcomes 70. This raises the intriguing possibility that the elevated IL-1α in our model may be an endogenous neuroprotective response that D-NAC appropriately spares. We also found that TNF-α was not significantly elevated in infected rabbits. This likely reflects the chronic nature of our model, as TNF-α typically peaks rapidly (within h) and then declines in the early stages of infection in other animal models and *in vitro* studies of TB meningitis 18, 71. While our overall findings suggest a targeted immunomodulatory effect, future studies are warranted to elucidate the precise downstream molecular mechanisms through which D-NAC modulates microglial activity to exert its neuroprotective effects.

Our studies have some limitations. Direct *M. tuberculosis* intraventricular inoculation does not model the initial brain dissemination process, but it does provide reliable brain pathology that mimics many features seen in patients with TB meningitis 11, 14, 15, 38. D-NAC was tested as a monotherapy to assess whether immunomodulatory agents would increase bacterial burden. While combination therapy with standard antimicrobials is the next necessary step, dendrimers are quickly excreted by the kidneys 72, reducing the risk of additional liver toxicity associated with many antimicrobials used in TB meningitis (e.g., rifampin). As mentioned earlier, our focus on a two-week treatment period targets the critical therapeutic window where mortality is highest and early optimization of therapy is clinically paramount 60, 61, 73. Nonetheless, future studies combining D-NAC with antimicrobials over longer durations are necessary to evaluate potential synergistic effects and long-term benefits before clinical translation. While the present study evaluates dendrimer-based noninvasive imaging and dendrimer-mediated therapeutic delivery as separate components, the use of the same dendrimer backbone provides a strong foundation for the future development of a fully integrated theranostic workflow.

## Conclusions

In summary, our study establishes dendrimer as a highly effective theranostic platform for visualizing and treating neuroinflammation in TB meningitis. We provided proof-of-concept data showing that a radiolabeled dendrimer (^124^I-dendrimer) can noninvasively identify sites of neuroinflammation. As a therapeutic agent, D-NAC selectively targeted activated microglia, decreasing neuroinflammation and cytokine levels while improving myelination, BBB integrity, and neurological outcomes. Importantly, the robust therapeutic effect of microglia-targeted therapy further validates the central role of activated microglia in TB meningitis pathology. This study highlights D-NAC as a promising host-directed therapy for TB meningitis and supports the clinical potential of dendrimer nanoplatforms for diagnosing and treating CNS infections.

## Supplementary Material

Supplementary figures and tables.

Supplementary source data file.

## Data Availability

All data are available in the main text or the supplementary materials. Source data are provided with this paper.

## Abbreviations

BBB: blood-brain barrier
BCA: bicinchoninic acid
BSL-3: biosafety level-3
CDCl_3_: deuterated chloroform or chloroform-d
CHCl_3_: chloroform
CFU: colony-forming units
CH_2_Cl_2_: dichloromethane
CNS: central nervous system
COVID-19: coronavirus disease 2019
CSF: cerebrospinal fluid
CT: computed tomography
CuAAC: copper(I)-catalyzed azide-alkyne cycloaddition
D-Cy5: dendrimer-Cy5
DIPEA: *N*,*N*-diisopropylethylamine
DMAP: 4-dimethylaminopyridine
DMF: *N,N*-dimethylformamide
DMSO: dimethyl sulfoxide
D-NAC: dendrimer-*N*-acetyl cysteine
EDC: 1-ethyl-3-(3-dimethylaminopropyl)carbodiimide
^18^F-FDG: ^18^F-fluorodeoxyglucose
g: gram
GEE: generalized estimating equations
GFAP: glial fibrillary acidic protein
h: hour
HIV: human immunodeficiency virus
^1^H NMR: proton nuclear magnetic resonance
HOAc: acetic acid
HPLC: high-performance liquid chromatography
HU: Hounsfield unit
Hz: hertz
H_2_O: water
Iba-1: ionized calcium-binding adapter molecule 1
%ID: percent injected dose
IL: interleukin
IQR: interquartile range
J: coupling constant
kDa: kilodalton
kg: kilogram
LTA4H: leukotriene A4 hydrolase
MBq: megabecquerel
MeOH: methanol
mg: milligram
MHz: megahertz
MIP-1β: macrophage inflammatory protein-1β
min: minute
mL: milliliter
mmol: millimole
MMP-9: matrix metalloproteinase-9
MRI: magnetic resonance imaging
N_2_: nitrogen
NAC: *N*-acetyl cysteine
Na^124^I: sodium iodide I-124
Na_2_SO_4_: sodium sulfate
nm: nanometer
O_2_: oxygen
PAMAM: polyamidoamine
PBS: phosphate buffered saline
PEG: polyethylene glycol
PET: positron emission tomography
PFA: paraformaldehyde
pg: picogram
ppm: parts per million
radio-HLPC: radio-high-performance liquid chromatography
RT: room temperature
s: second
SD: standard deviation
STB: *N*-succinimidyl 3-(tri-n-butylstannyl)benzoate
SUV: standard uptake value
TB: tuberculosis
TB meningitis: tuberculous meningitis
TBS: tris-buffered saline
Th17: T helper 17
TNF: tumor necrosis factor
TSPO: translocator protein
t_1/2_: half-life
µg: microgram
µL: microliter
µm: micrometer
VOI: volume of interest
WHO: World Health Organization
xCT: cysteine-glutamate antiporter
