# Supplementary material for: Host-directed nanotherapy for the treatment and imaging of tuberculous meningitis
Source: Theranostics. 2026 Mar 3;16(10):5105–24. doi: 10.7150/thno.125729 (PMC13080335; doi:10.7150/thno.125729)
Supplement: Supplementary file 1 — Supplementary figures and tables. [file thnov16p5105s1.pdf]

Host-directed nanotherapy for the treatment and imaging of tuberculous meningitis

Elizabeth W. Tucker[1,2,3]*†, John Kim[1,2,3]†, Clara Erice[1,2,3]†, Anjali Sharma[4#]†, Nerketa N. L. Damiba[1,2,3], Alvaro A. Ordonez[1,2,5], Javier Allende Labastida[3,4], Nirnath Sah[1,4], Filipa Mota[1,2,5], Patricia de Jesus[1,2,5], Rhea Saini[3], Kelly F. Schiaffino[1,2,5], Sanjay K. Jain[1,2,5##], Rangaramanujam M. Kannan[4], Sujatha Kannan[3,4]

Affiliations

[1]Center for Infection and Inflammation Imaging Research, Johns Hopkins University School of Medicine, Baltimore, MD 21287, USA.

[2]Center for Tuberculosis Research, Johns Hopkins University School of Medicine, Baltimore, MD 21287, USA.

[3]Department of Anesthesiology and Critical Care Medicine, Johns Hopkins University School of Medicine, Baltimore, MD 21287, USA.

[4]Center for Nanomedicine, Johns Hopkins University School of Medicine, Baltimore, MD 21287, USA.

[5]Department of Pediatrics, Johns Hopkins University School of Medicine, Baltimore, MD 21287, USA.

[#]Current affiliation and address for Anjali Sharma: Department of Chemistry, College of Arts and Sciences, Washington State University, Pullman, WA 99164, USA.

[##]Current affiliation and address for Sanjay K. Jain: Department of Pediatrics and Cincinnati Children's Center for Molecular Imaging and Precision Medicine, Cincinnati Children’s Hospital Medical Center, Cincinnati, OH 45229, USA.

†These authors contributed equally to this work

*Corresponding author:

Elizabeth W. Tucker

Phone: 410-955-9408

Email: etucker9@jh.edu

SUPPLEMENTARY TEXT

Methods

Neurobehavioral testing:

The automated behavior component calculated Ethovision XT (Noldus) scores from uninfected, age- and litter-matched rabbits, which were used to establish the mean and standard deviation (SD) for each parameter. The degree of deviation (i.e., number of SDs from the mean) was then used to score all rabbits. Each value received a z-score using the following formula:

$$\text{Z-score} = (\text{value} - \bar{X}) / \sigma$$

where $\bar{X}$ is the uninfected mean, and σ is the uninfected SD. The z-score point allocation is listed in **Supplementary Table 1**.

Additionally, inter-rater reliability measures for the manual behavior component were calculated for the 89 videos scored by two independent raters (J.K. and R.S.). These included the observed agreement rate and the quadratically weighted Cohen's κ for each behavioral item. Two components of the manual scoring showed perfect agreement, with 100% observed agreement across the two independent raters for clinical seizure and eye opening. Among items with some disagreement, most showed high concordance. For example, coat maintenance, head tilt, and hind limb paresis severity exhibited high concordance, with observed agreement rates of 99.3%, 90.4%, and 63.0%, and quadratically weighted κ values of 0.80, 0.63, and 0.74, respectively. Balance and front limb paresis severity had high observed agreement (89.6% and 91.1%) but low κ values (near zero). This aligns with the known prevalence-related κ paradox, where high observed agreement occurs when one outcome is common but κ is low. Agreement was lower for more subjective items, such as hop and limb paresis, with observed agreement of 39.3% and 66.7%, and κ values of 0.28 and 0.18. Additionally, we created a composite behavioral score for each video by summing all item scores of the manual component, then

calculated the Intraclass Correlation Coefficient (ICC) between the two raters. The ICC (Model 3, single measurement) was 0.76, indicating ‘good’ reliability, suggesting that about three-quarters of the variability in manual scores was due to fundamental differences between subjects rather than scoring differences between raters.

SUPPLEMENTARY FIGURES

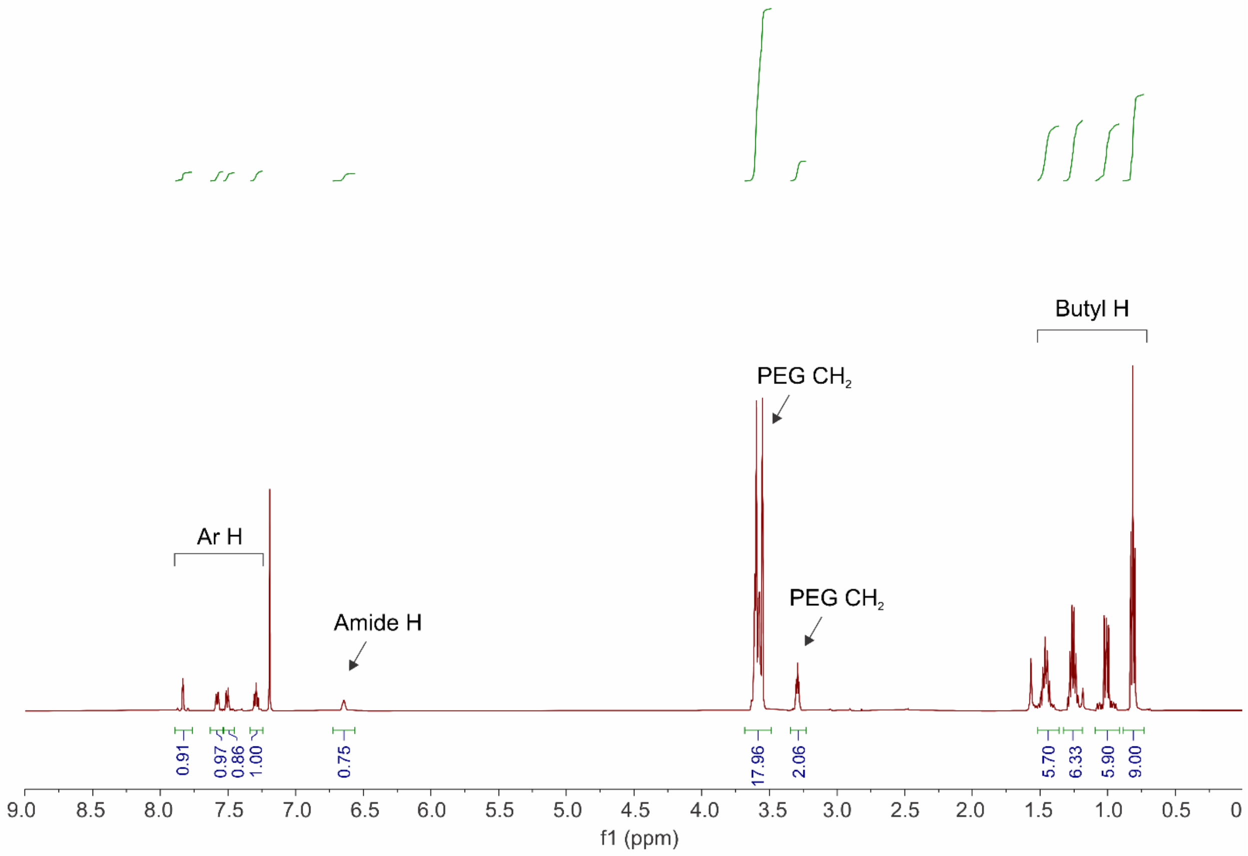

Figure S1. Structural determination of precursor compound 2. ^{1}H NMR of compound **2** (chloroform-d [$CDCl_3$], 500MHz) shows PEG *H*.

A

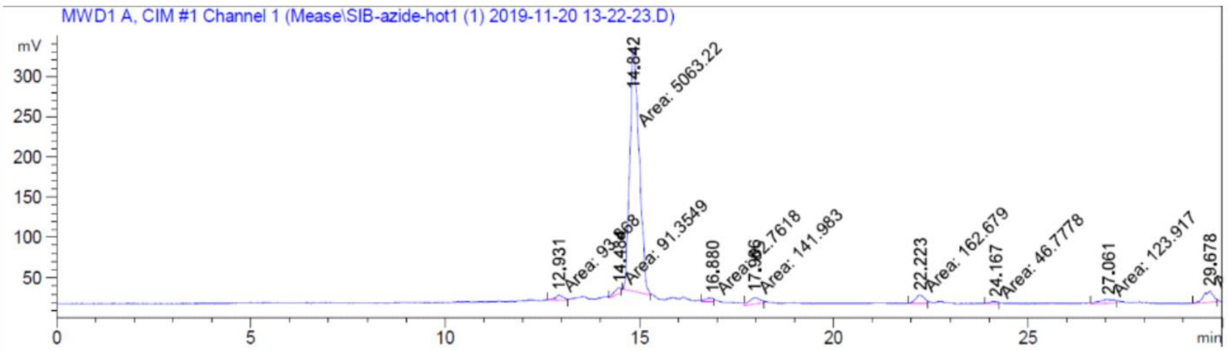

Peak #	RetTime [min]	Type	Width [min]	Area [mV*s]	Height [mV]	Area %
1	12.931	MM	0.2612	93.86805	5.98853	1.5575
2	14.484	MM	0.2040	91.35491	7.46246	1.5158
3	14.842	MM	0.2797	5063.22168	301.75452	84.0104
4	16.880	MM	0.2258	62.76178	4.63191	1.0414
5	17.966	MM	0.2868	141.98323	8.25239	2.3558
6	22.223	MM	0.2655	162.67896	10.21103	2.6992
7	24.167	MM	0.2368	46.77782	3.29287	0.7762
8	27.061	MM	0.4557	123.91729	4.53215	2.0561
9	29.678	MM	0.2876	240.33691	13.93003	3.9877

B

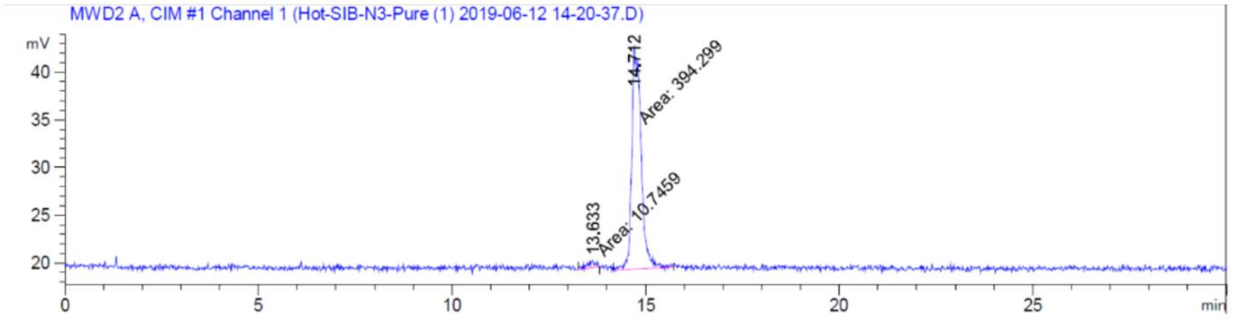

Peak #	RetTime [min]	Type	Width [min]	Area [mV*s]	Height [mV]	Area %
1	13.633	MM	0.2198	10.74594	8.14833e-1	2.6530
2	14.712	MM	0.2774	394.29868	23.68610	97.3470

Figure S2. Radio-high-performance liquid chromatography (radio-HPLC) trace of compound 3. (**A** and **B**) Radio-HPLC trace of compound **3** (**A**) before and (**B**) after purification.

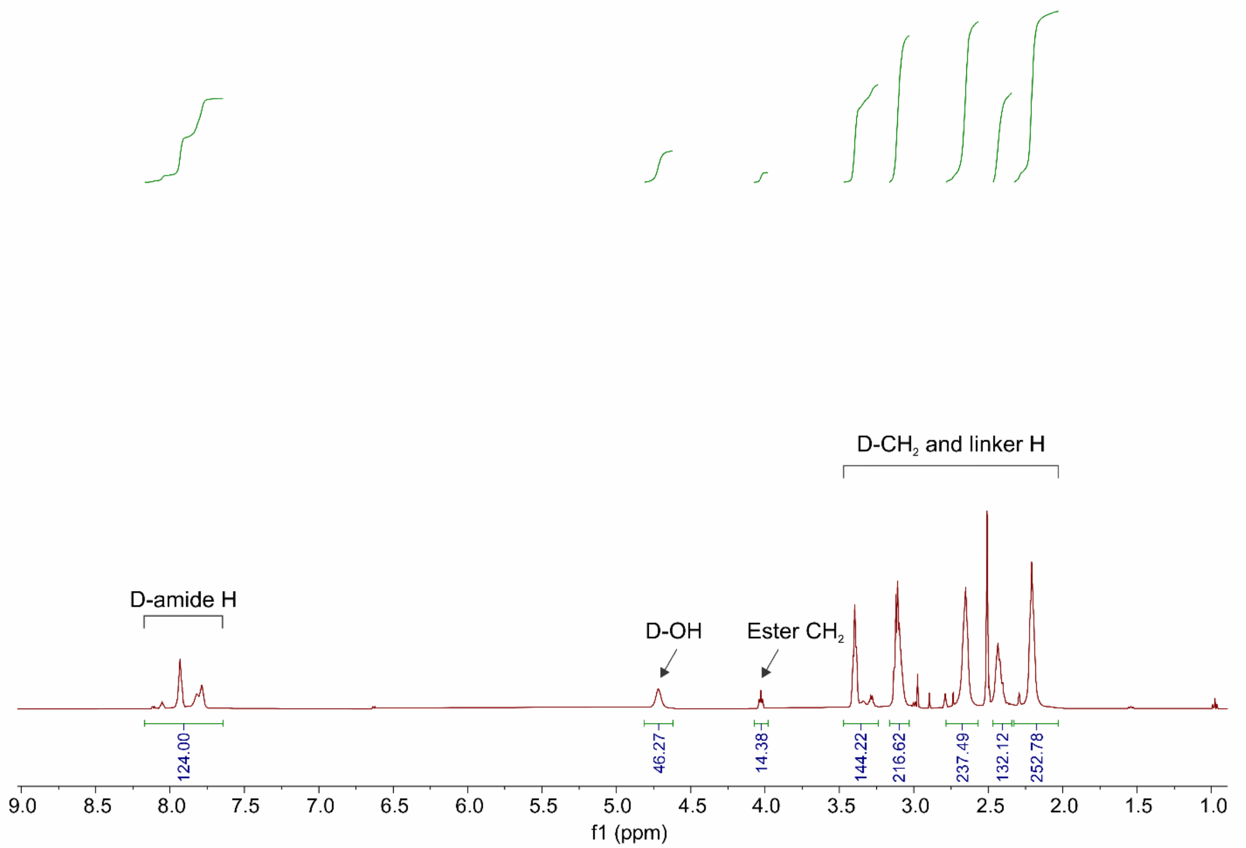

Figure S3. Structural determination of precursor compound 5. ^{1}H NMR of compound **5** (dimethyl sulfoxide [DMSO]-*d6*, 500MHz). Abbreviations: D, dendrimer.

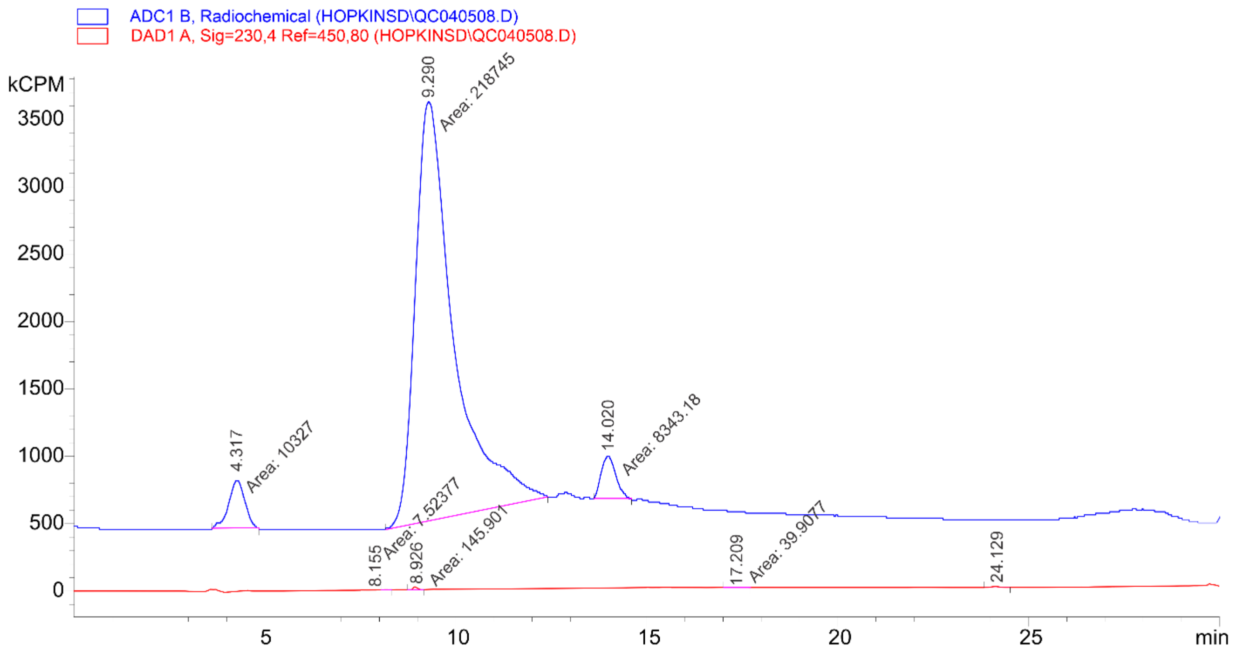

Signal 1: ADC1 B, Radiochemical

Peak #	RetTime [min]	Type	Width [min]	Area [kCPM*s]	Height [kCPM]	Area %
1	4.317	MM	0.4925	1.03270e4	349.48782	4.3498
2	9.290	MM	1.1706	2.18745e5	3114.43042	92.1361
3	14.020	MM	0.4441	8343.18164	313.12924	3.5142

Figure S4. Radio-HPLC trace of compound **6** (92% purity).

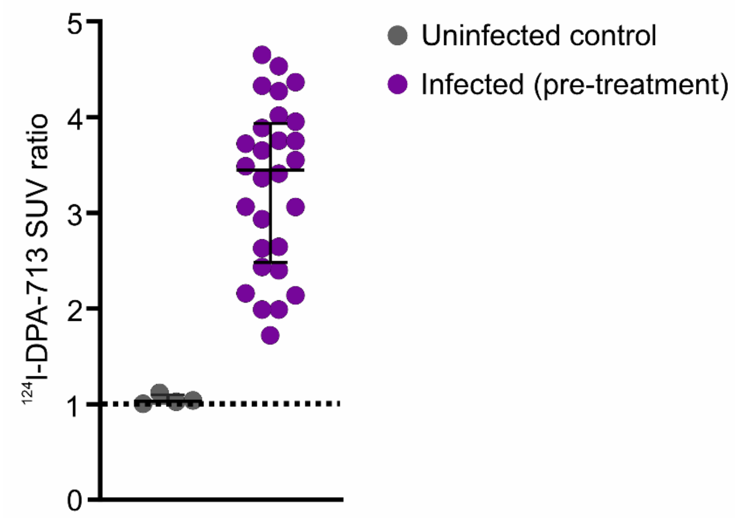

Figure S5. Baseline ^{124}I-DPA-713 signal in the brains of uninfected control and infected (pre-treatment) rabbits. The SUV ratio ~1 (dotted black line) in uninfected control rabbits (i.e., injected with PBS) shows minimal left–right hemispheric variability, unlike the increased signal in the brain lesion seen in infected rabbits prior to treatment. Each dot represents a volume of interest (VOI). Data include the following animals: uninfected control (*n* = 2, with 2 VOIs per animal, totaling 4 VOIs) and infected pre-treatment (*n* = 11, with 2-3 VOIs per animal, totaling 28 VOIs). Data are shown as median ± IQR. The source data are included in a Source Data File.

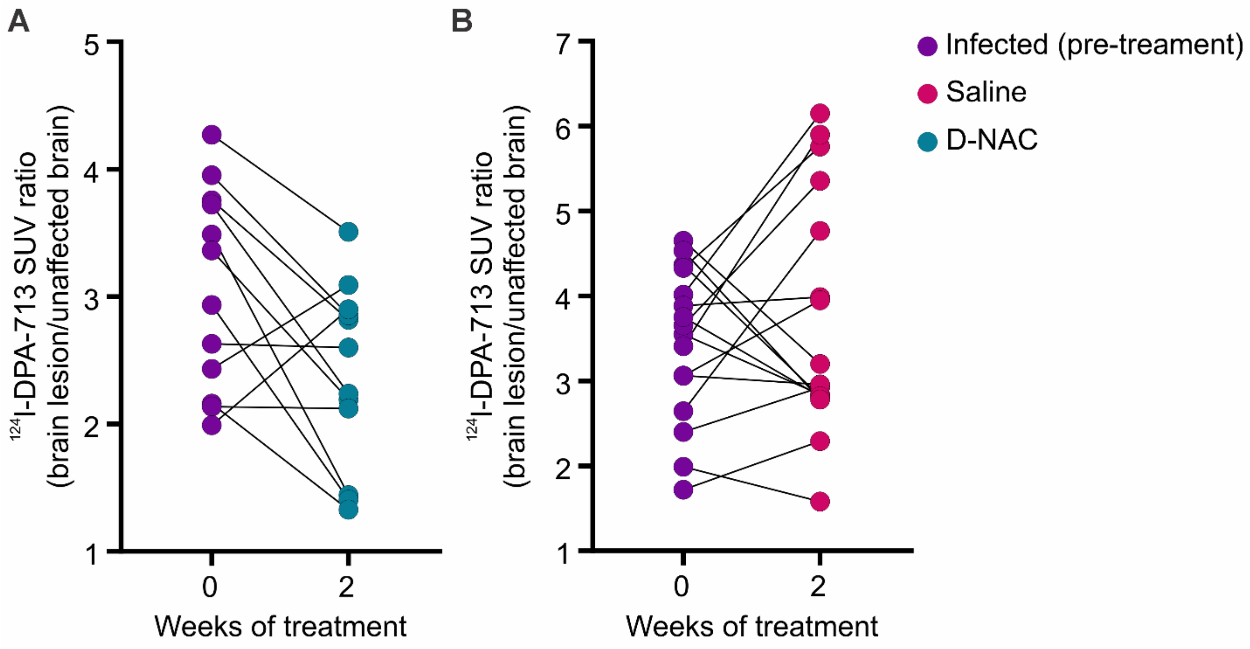

Figure S6. Individual ^{124}I-DPA-713 VOIs over time. (**A** and **B**) PET-derived SUV ratios in infected rabbits treated with (**A**) D-NAC and (**B**) saline. Each dot represents a VOI. Data include the following animals: D-NAC-treated (*n* = 5, with 2-3 VOIs per animal, totaling 12 VOIs) and saline-treated (*n* = 6, with 2-3 VOIs per animal, totaling 16 VOIs). The source data are included in a Source Data File.

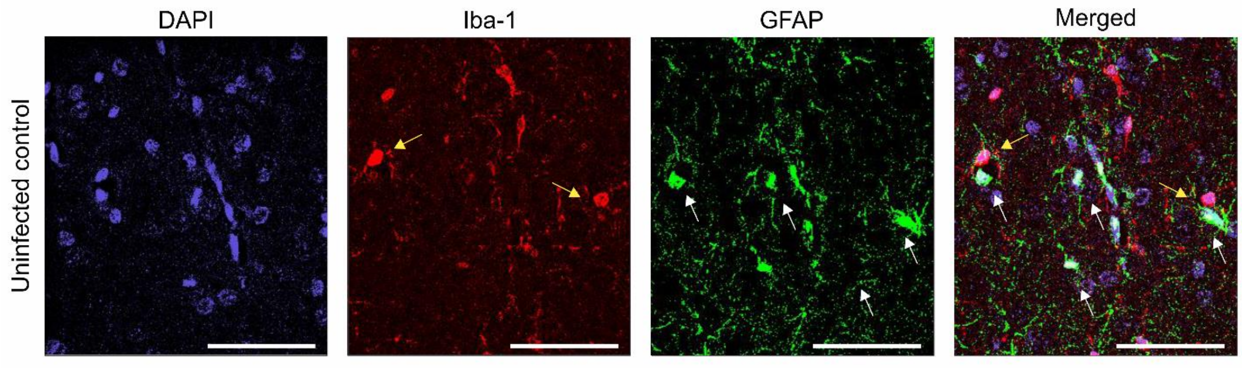

Figure S7. Uninfected control brain immunohistochemistry. Baseline microglia and astrocyte density in uninfected control rabbit brain. Representative confocal images of microglia (Iba-1, yellow arrows) and astrocytes (GFAP, white arrows) co-localized with DAPI (nuclear stain). Scale bar = 50 µm. Abbreviations: GFAP: glial fibrillary acidic protein; Iba-1: ionized calcium-binding adapter molecule 1.

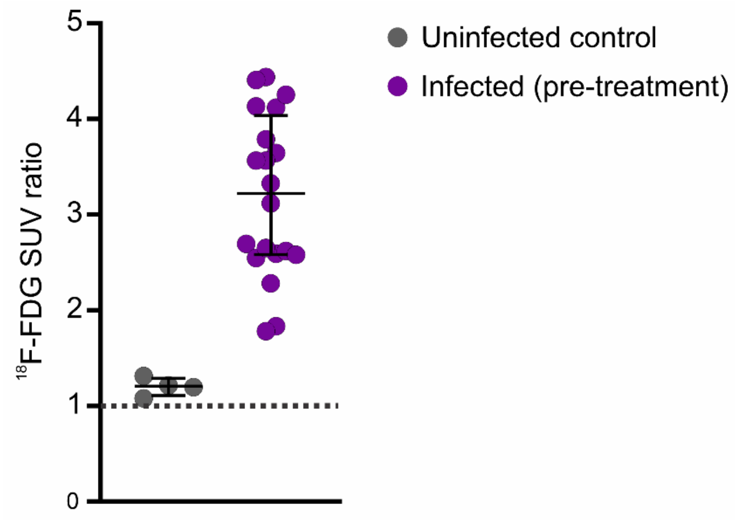

Figure S8. Baseline ^{18}F-FDG signal in the brains of uninfected control and infected (pre-treatment) rabbits. The SUV ratio ~1 (dotted black line) in uninfected control rabbits (i.e., injected with PBS) shows minimal left–right hemispheric variability, unlike the increased signal in the brain lesion seen in infected rabbits prior to treatment. Each dot represents a VOI. Data include the following animals: uninfected control (*n* = 2, with 2 VOIs per animal, totaling 4 VOIs) and infected pre-treatment (*n* = 8, with 2-3 VOIs per animal, totaling 20 VOIs). Data are shown as median ± IQR. The source data are included in a Source Data File.

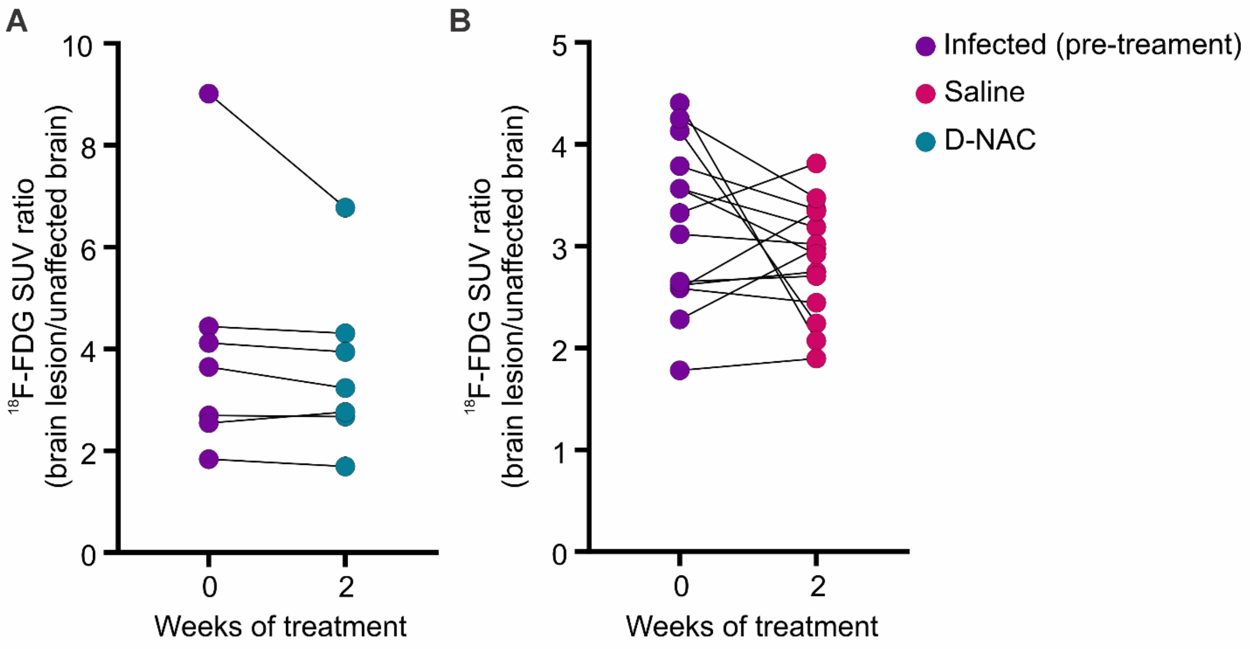

Figure S9. Individual ^{18}F-FDG VOIs over time. (**A** and **B**) PET-derived SUV ratios in infected rabbits treated with (**A**) D-NAC and (**B**) saline. Each dot represents a VOI. Data include the following animals: D-NAC-treated (*n* = 3, with 2-3 VOIs per animal, totaling 7 VOIs) and saline-treated (*n* = 5, with 2-3 VOIs per animal, totaling 14 VOIs). The source data are included in a Source Data File.

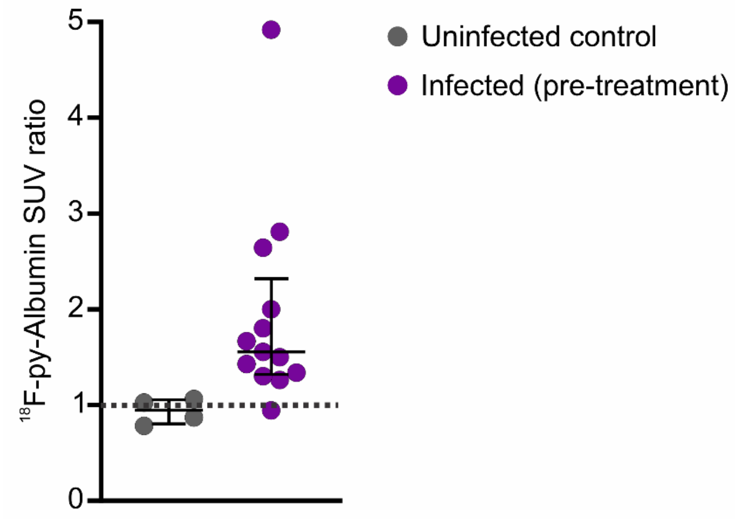

Figure S10. Baseline ^{18}F-py-albumin signal in the brain of uninfected control and infected (pre-treatment) rabbits. The SUV ratio ~1 (dotted black line) in uninfected control rabbits (i.e., injected with PBS) shows minimal left–right hemispheric variability, unlike the increased signal in the brain lesion seen in infected rabbits prior to treatment. Each dot represents a VOI. Data include the following animals: uninfected control (*n* = 2, with 2 VOIs per animal, totaling 4 VOIs) and infected pre-treatment (*n* = 5, with 2-3 VOIs per animal, totaling 13 VOIs). Data are presented as median ± IQR. The source data are included in a Source Data File.

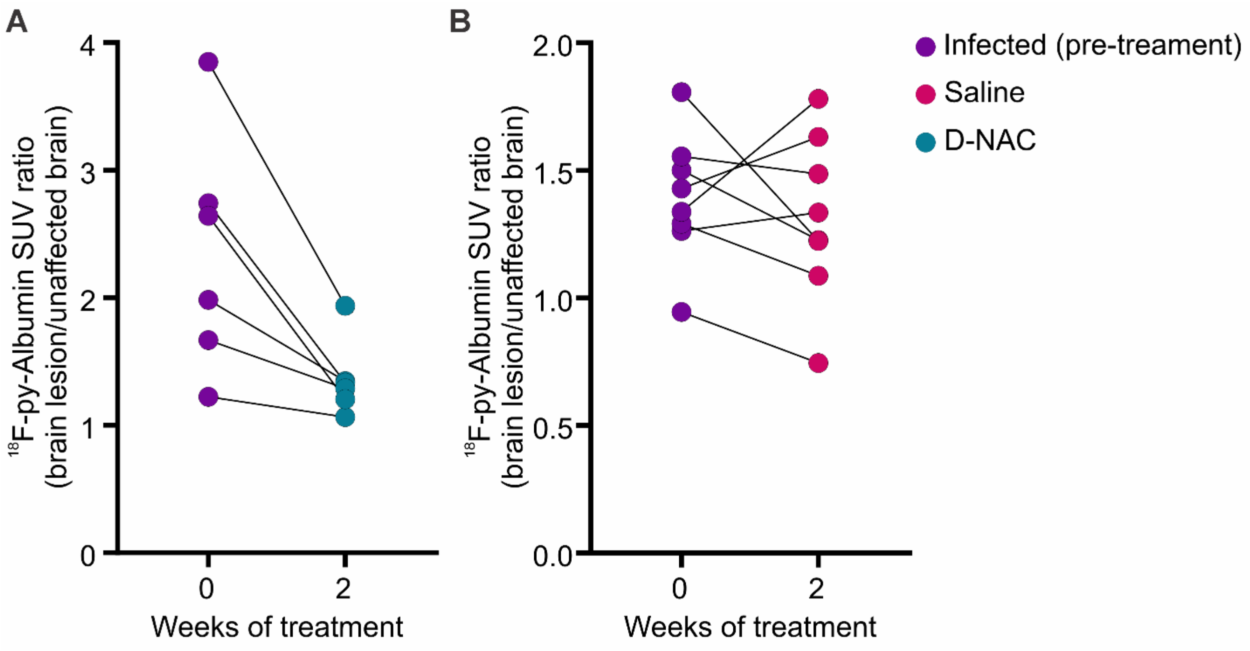

Figure S11. Individual ^{18}F-py-albumin VOIs over time. (**A** and **B**) PET-derived SUV ratios in infected rabbits treated with (**A**) D-NAC and (**B**) saline. Each dot represents a VOI. Data include the following animals: D-NAC-treated (*n* = 2, with 3 VOIs per animal, totaling 6 VOIs) and saline-treated (*n* = 3, with 2-3 VOIs per animal, totaling 8 VOIs). The source data are included in a Source Data File.

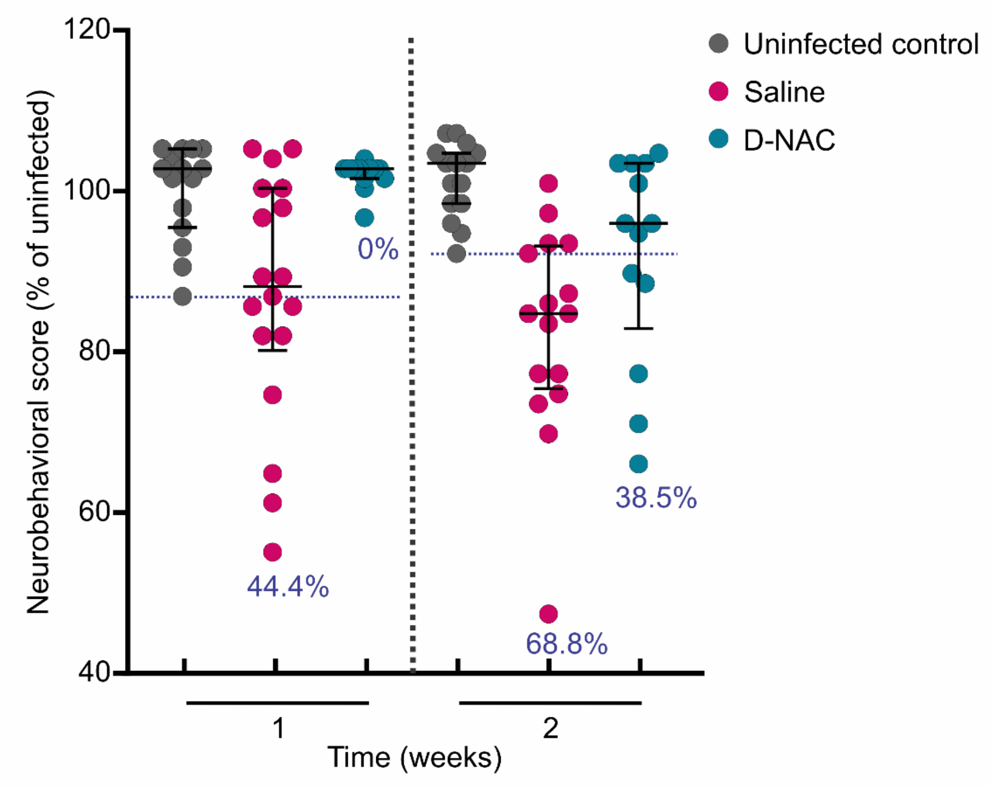

Figure S12. Proportion of neurobehavioral scores below the score of uninfected control, age-matched littermates. Longitudinal neurobehavioral scores (% of uninfected control score) are shown after one and two weeks of treatment (weeks separated by a dotted black line). Each dot represents an individual score. A dotted blue line indicates the lowest neurobehavioral score of uninfected control, age-matched littermates. The percentages of scores below this threshold are shown in blue: saline-treated week 1 was 44.4% (8/18 scores) and week 2 was 68.8% (11/16 scores); D-NAC-treated week 1 was 0% (0/12 scores) and week two 38.5% (5/13 scores). Data include the following animals, each with 1-2 scores: uninfected control (*n* = 8), saline-treated (*n* = 9), and D-NAC-treated (*n* = 7). Data are presented as median ± IQR. The source data are included in a Source Data File.

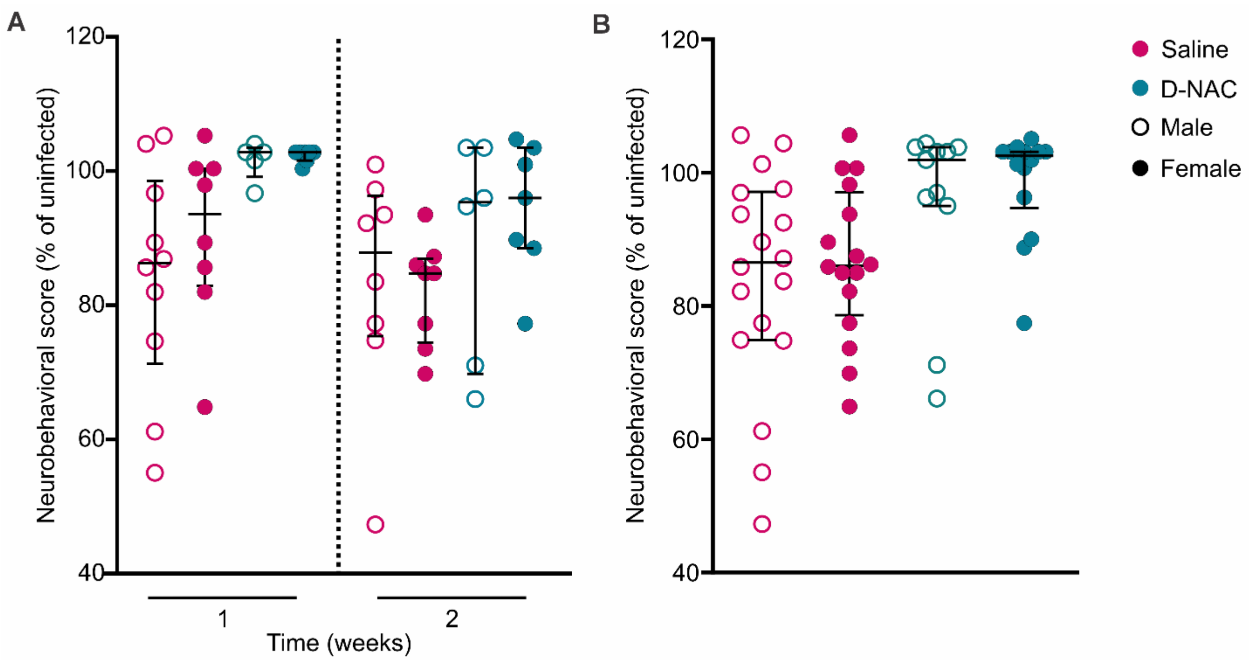

Figure S13. Neurobehavioral scores based on sex. (**A** and **B**) Longitudinal neurobehavioral score shown by sex and treatment group, plotted by (**A**) weeks of treatment (separated by dotted vertical black line) and (**B**) pooled across time points. Each dot represents an individual score. Data include the following animals, each with 1-2 scores: male saline-treated (*n* = 5), female saline-treated (*n* = 4), male D-NAC-treated (*n* = 3), and female D-NAC-treated (*n* = 4). Data are presented as median ± IQR. The source data are included in a Source Data File.

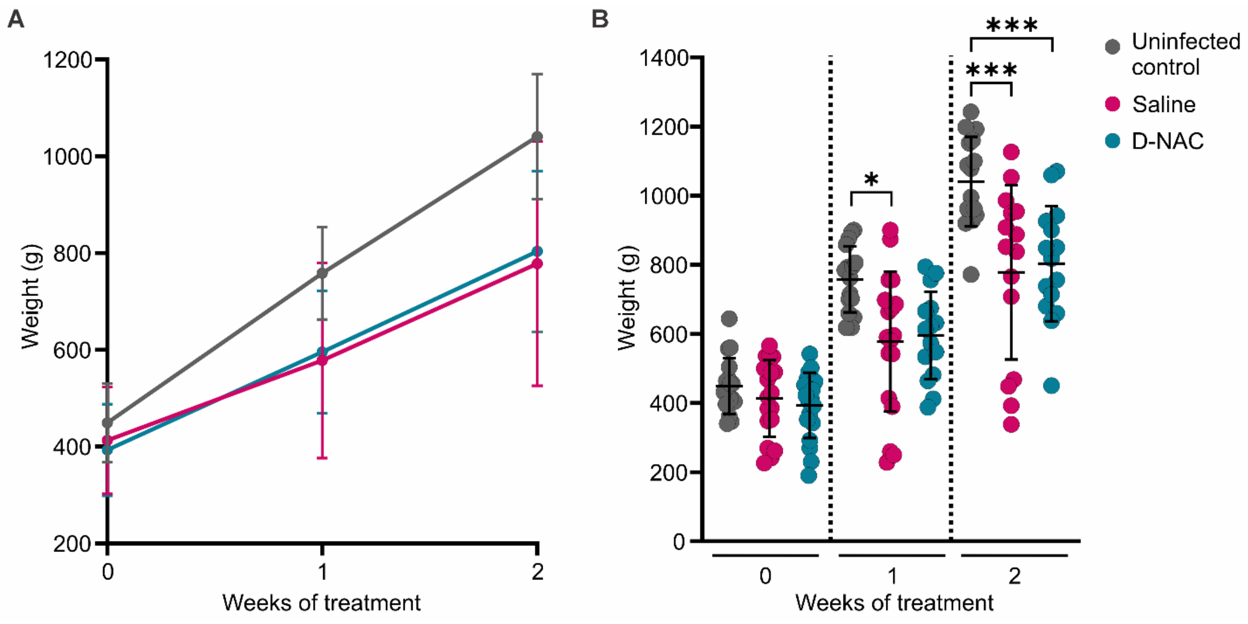

Figure S14. Weight changes over time. (**A** and **B**) Body weight changes (g) during treatment are shown as (**A**) mean change and (**B**) individual data points over time. Time points are separated by dotted vertical black lines. Data include the following numbers of animals per time point: uninfected control (*n* = 16-17), saline-treated (*n* = 15-18), and D-NAC-treated (*n* = 15-19). Data are presented as mean ± standard deviation (SD). Statistical comparisons were made using ANOVA with Tukey's multiple comparisons. **P* < 0.05, ****P* < 0.001. The source data are included in a Source Data File.

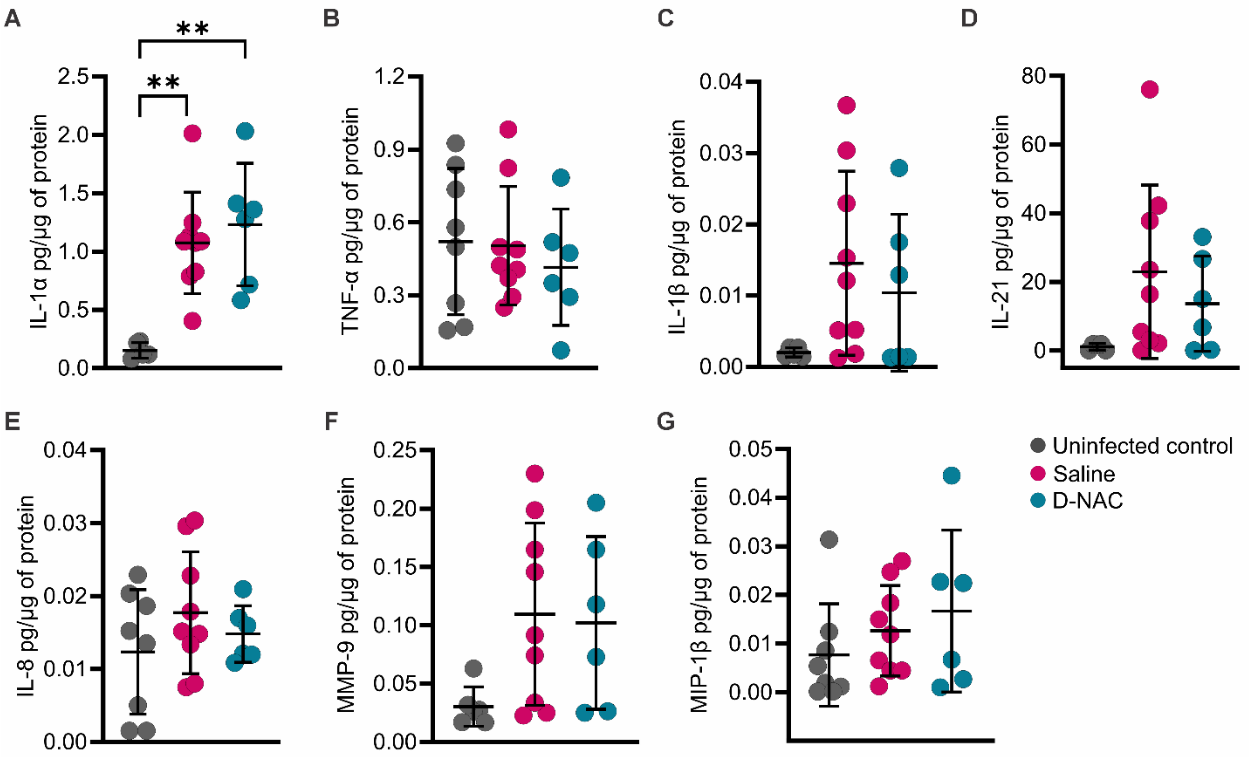

Figure S15. CSF inflammatory marker analysis. (**A** and **G**) Quantification of CSF (**A**) IL-1α, (**B**) TNF-α, (**C**) IL-1β, (**D**) IL-21, (**E**) IL-8, (**F**) MMP-9, and (**G**) MIP-1β after two weeks of treatment. Data include the following animals: uninfected control (n = 5-8), saline-treated (n = 9), and D-NAC-treated (n = 6). Data are presented as mean ± SD. Statistical comparisons were performed using ANOVA with Tukey's multiple comparisons. **P < 0.01. The source data are included in a Source Data File.

SUPPLEMENTARY TABLES

Table S1. Z-score point allocation for Ethovision XT (Noldus) neurobehavioral score calculation.

**Z-score**	**Points**
Within ± 1	3
Within ± 2	2
Within ± 3	1
Beyond ± 3	0

Table S2. Automated Noldus neurobehavioral scoring component. Each parameter earned 0-3 points for a total of 21 possible points (perfect score).

**Parameter**	**Definition**
Mean Velocity	Velocity of the body center point.
Distance Traveled	Total distance moved of rabbit center-point within 2-minute (min) video recording.
Mean Absolute Turn Angle	The change in moving direction of the center point. Absolute turn angle does not take the direction of turning into account (clockwise or counterclockwise). For example, stereotypy is characterized by quick changes in motion direction (turn angle).
Mean Absolute Angular Velocity	The change in moving direction of the center point per unit of time. For example, measuring the tendency of an animal to turn to a specific direction or object.
Cumulative Duration of Movement	During the 2-min recording, how much time did the animal spend moving (defined as movement of the body center-point from one location to another).
Cumulative Duration of Mobility	The duration over which the entire area is detected as an animal changes, even if the center point remains the same.
Cumulative Duration of Normal Body Posture (Elongation)	The duration for which the animal exhibits "normal" state of posture/elongation (below 35% contraction and 90% stretch).

Table S3. Manual neurobehavioral scoring component. Each parameter score was added for a total of 20 possible points (perfect score).

**Parameter**	**Definition**	**Max Points**
Clinical Seizure	Not observed (1 point)<br>Observed (0 point)	1
Head Tilt Severity	Not observed (2 points)<br>Moderate head tilt of ≤45° (1 point)<br>Severe head tilt of ≥46° (0 point)	2
Limb Paresis	Not observed (2 points)<br>Monoparesis (1 point)<br>Paraparesis (0 point)	2
Hind Limb Paresis Severity	Not observed (3 points)<br>Occasional instances of butterfly hindlimb(s) of ≤45° (2 points)<br>Moderate butterfly of hindlimb(s) of ≤45° (1 point)<br>Severe butterfly of hindlimb(s) of ≥46° (0 point)	3
Front Limb Paresis Severity	Not observed (2 points)<br>Moderate (front limb paresis observed ≤50% of time) (1 point)<br>Severe front limb paresis observed ≥51% of time) (0 point)	2
Eye Opening	Both Eyes (2 points)<br>One eye (1 point)<br>None (0 point)	2
Hop (A successful "hop" is a movement when both hindlimbs move together. The "hop" doesn't have to be completed with strength or large distance)	Hop 76-100% of movements (5 points)<br>Hop 51-75% of movements (4 points)<br>Hop 26-50% of movements (3 points)<br>Hop 0-25% of movements (2 points)<br>No hops (all movements are walking/crawling) (1 point)<br>Both limbs disabled; hop impossible (0 point)	5
Balance	Normal Balance (3 points)<br>Unsteady (at least one fall/flip over) (2 points)<br>Falls/Flips <50% of time recorded (1 point)<br>Falls/Flips 50-100% of time (0 point)	3
